# Mg‐doped α‐Ga_2_O_3_ Nanorods for the Construction of Photoelectrochemical‐Type Self‐Powered Solar Blind UV Photodetectors and Underwater Imaging Application

**DOI:** 10.1002/advs.202413074

**Published:** 2025-02-26

**Authors:** Xin Zhou, Lijuan Ye, Lai Yuan, Dan Zhang, Hong Zhang, Di Pang, Yan Tang, Honglin Li, Wanjun Li, Heping Zeng

**Affiliations:** ^1^ Guangyang Bay Laboratory Chongqing Institute for Brain and Intelligence Chongqing 400064 China; ^2^ College of Physics and Electronic Engineering Chongqing Normal University Chongqing 401331 China; ^3^ Chongqing Key Laboratory of Precision Optics Chongqing Institute of East China Normal University Chongqing 401120 China; ^4^ State Key Laboratory of Precision Spectroscopy East China Normal University Shanghai 200241 China

**Keywords:** Ga_2_O_3_, Mg‐doping, photoelectrochemical, solar‐blind photodetector, underwater imaging

## Abstract

Underwater imaging technologies are increasingly crucial for environmental monitoring and resource exploration. However, the development of advanced photodetectors for such applications faces significant challenges, including interference from ambient visible and infrared light, adaptation to underwater environments, and cost‐effectiveness. Photoelectrochemical‐type solar‐blind photodetectors (PEC‐SBPDs) based on wide bandgap semiconductors have shown great promise in overcoming these challenges. Here, a novel approach to enhance the performance of α‐Ga_2_O_3_‐based PEC‐SBPDs is presented for underwater imaging through Mg‐doping. By employing a low‐cost hydrothermal synthesis technique, Mg‐doped α‐Ga_2_O_3_ nanorod arrays are fabricated, which induces the formation of V_O_‐Mg_Ga_ complexes that enhances the interfacial catalytic activity and improves the transport of photogenerated carriers. The optimized PEC‐SBPDs exhibits a remarkable 435% increase in photocurrent response compared to undoped α‐Ga_2_O_3_, with a peak responsivity of 34.54 mA W^−1^. A 5 × 5 PEC‐SBPD array based on Mg‐doped α‐Ga_2_O_3_ nanorods is successfully demonstrated for underwater solar‐blind imaging, achieving clear and efficient imaging in challenging underwater conditions. This study not only highlights the superior performance of Mg‐doped α‐Ga_2_O_3_ in underwater environments but also opens new avenues for the development of high‐performance self‐powered photodetectors in imaging, sensing, and other related applications.

## Graphical abstract

A low‐cost hydrothermal technique is employed to optimize the synthesis of Mg‐doped α‐Ga_2_O_3_ nanorod arrays. Compared to undoped α‐Ga_2_O_3_ photodetectors, the photocurrent response is increased by 435%, with a peak responsivity of 34.54 mA W^−1^. Furthermore, a 5 × 5 PEC‐SBPD array based on Mg‐doped α‐Ga_2_O_3_ nanorod arrays is constructed for underwater imaging applications.

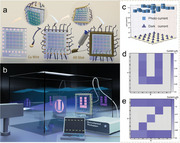

## 1 Introduction

Underwater imaging technology has become a crucial tool in environmental monitoring and resource exploration.^[^ ^1^ ^]^ However, developing advanced underwater imaging units, such as photodetectors, presents several challenges, including interference from visible and infrared light, adaptation to underwater environments, and cost‐effectiveness.^[^ ^2^ ^]^ In recent years, emerging PEC‐SBPDs have demonstrated significant advantages that effectively address these challenges:^[^ ^3^, ^4^, ^5^ ^]^ Natural light shielding: the solar‐blind deep ultraviolet (DUV) band is naturally shielded by atmospheric absorption, preventing it from reaching ground level. This drastically reduces background light interference for the photodetectors. 2) Hydrophilicity for underwater adaptation: PEC‐SBPDs exhibit hydrophilic properties, allowing direct contact with seawater and electrolytes, enhancing their adaptability to underwater environments. 3) Simplified fabrication and low power consumption: their production process is straightforward, eliminating the need for complex lithography and packaging steps, which helps reduce costs. Furthermore, PEC‐SBPDs operate with low power requirements, further increasing their practical value.

PEC‐SBPDs, particularly those focusing on ultra‐wide bandgap semiconductors like AlGaN and Ga_2_O_3_, hold immense potential for applications in optical communication, logic gates, synaptic devices, imaging systems, and so on. The Sun research group has achieved significant breakthroughs in the PEC‐SBPDs field, including the first‐ever construction of a p‐AlGaN/n‐GaN heterojunction photodetector capable of generating bipolar photocurrents.^[^ ^2^, ^6^ ^]^ This innovation has been successfully applied in underwater encrypted optical communication and logic gate designs.^[^ ^7^, ^8^ ^]^ Additionally, they have leveraged GaN‐based nanowire *p‐n* junctions to achieve biomimetic vision and precise blood glucose analysis in neuromorphic synaptic devices and biosensors.^[^ ^9^, ^10^ ^]^ Meanwhile, the Guo group has advanced Ga_2_O_3_‐based PEC‐SBPDs by introducing bipolar functionality through Cu_2_O/Ga_2_O_3_ heterostructures and titanium carbide surface modification, enabling their use in encrypted optical communication and logic gate applications.^[^ ^11^, ^12^, ^13^ ^]^ Our research group has further enhanced the performance of Ga_2_O_3_ PEC‐SBPDs by constructing α‐Ga_2_O_3_@α‐Al_2_O_3_ nanorod array heterojunctions and fine‐tuning V_O_ in amorphous Ga_2_O_3_ thin films. These innovations have enabled the successful development of PEC‐SBPD arrays for solar‐blind imaging.^[^ ^14^, ^15^ ^]^ Compared to conventional solid‐state array imaging technologies, Ga_2_O_3_‐based PEC‐SBPD arrays offer several advantages, including simplified electrode fabrication, unaffected photosensitive regions, and cost‐effectiveness. These advancements open new perspectives and opportunities for the evolution of PEC photodetection technologies.

Recently, significant progress has been made in the field of Ga_2_O_3_‐based PEC‐SBPDs.^[^ ^16^ ^]^ Among them, amorphous Ga_2_O_3_ (a‐Ga_2_O_3_), β‐Ga_2_O_3_, and α‐Ga_2_O_3_ have emerged as key photoanode materials, demonstrating tremendous application potential. For a‐Ga_2_O_3_ PEC‐SBPDs, our research group successfully constructed devices using 3D conductive substrates such as carbon fiber paper,^[^ ^17^ ^]^ carbon cloth,^[^ ^18^ ^]^ and silver nanowires.^[^ ^19^ ^]^ These designs not only provide an efficient network for photogenerated carrier transport but also significantly heighten detectors’ responsivity.^[^ ^15^ ^]^ For β‐Ga_2_O_3_ PEC‐SBPDs, researchers have achieved efficient PEC conversion by leveraging its stable integration on high‐temperature‐resistant substrates such as silicon^[^ ^20^ ^]^ and GaN.^[^ ^21^ ^]^ As for α‐Ga_2_O_3_ PEC‐SBPDs, its low‐cost, highly efficient hydrothermal synthesis process has garnered considerable attention. α‐Ga_2_O_3_ can be obtained via the generation of a GaOOH precursor followed by low‐temperature annealing.^[^ ^22^ ^]^ This fabrication approach enables the controlled synthesis of α‐Ga_2_O_3_ nanostructure arrays and paves the way for constructing high‐performance PEC‐SBPDs. For example, Jiao and Guo's group successfully tuned carrier dynamics in PEC photodetectors by constructing heterojunctions of α‐Ga_2_O_3_ with γ‐Al2O3^[^ ^23^ ^]^ or Gu_2_O,^[^ ^24^ ^]^ achieving the successful fabrication of various nanorod‐based PEC‐SBPDs. However, compared to a‐Ga_2_O_3_ and β‐Ga_2_O_3_ PEC‐SBPDs, α‐Ga_2_O_3_‐based PEC‐SBPDs still face challenges such as high interfacial impedance and limited interfacial reaction activity.^[^ ^25^ ^]^ These limitations hinder the effective separation and transport of carriers, ultimately affecting overall device performance.^[^ ^26^ ^]^ Doping, as a classic semiconductor modification technique, not only directly influences the carrier concentration within the material but also induces new defects or complexes, enabling efficient regulation of the solid‐liquid interface.^[^ ^27^, ^28^, ^29^ ^]^ This approach holds great significance for the transfer of photogenerated carriers and solid‐liquid interfacial catalytic processes.

In this work, a low‐cost hydrothermal technique was employed to optimize the synthesis of Mg‐doped α‐Ga_2_O_3_ nanorod arrays, inducing the formation of V_O_‐Mg_Ga_ complexes. These complexes not only increased the concentration of V_O_, thereby enhancing interfacial catalytic activity but also elevated the donor energy level of V_O_, improving interfacial conductivity and facilitating carrier transport. As a result, Mg doping significantly enhanced the performance of the α‐Ga_2_O_3_ photodetector. Compared to undoped α‐Ga_2_O_3_ photodetectors, the photocurrent response was increased by 435%, with a peak responsivity of 34.54 mA W^−1^. Furthermore, a 5 × 5 PEC‐SBPD array based on Mg‐doped α‐Ga_2_O_3_ nanorod arrays was constructed for underwater imaging applications. This array achieved clear imaging in the solar‐blind UV region underwater in a straightforward, efficient manner. These findings not only demonstrate the exceptional performance of Mg‐doped α‐Ga_2_O_3_ materials in underwater environments but also open new opportunities for applications in imaging, sensing, and related fields.

## 2 Results and Discussion

**Figure 1:**
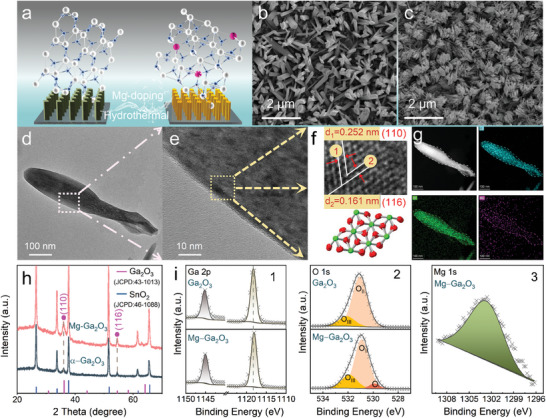
a) Schematic diagram of Mg‐doped α‐Ga_2_O_3_ nanorods. b,c) SEM images of α‐Ga_2_O_3_ and Mg‐Ga_2_O_3_ nanorods, respectively. d) TEM image of an individual Mg‐Ga_2_O_3_ nanorod. e) HR‐TEM image of the selected region. f) Inverse FFT image of the specific region. g) EDS images of an individual nanorod. h) XRD pattern of α‐Ga_2_O_3_ and Mg‐Ga_2_O_3_. i) XPS elemental peak analysis. (i–1) The XPS peak fitting of Ga 2*p*. (i‐2) The XPS peak fitting of O 1*s*. (i‐3) Peak fitting plots of elemental Mg.

**Figure** **1**a illustrates the schematic diagram of Mg‐doped α‐Ga_2_O_3_ nanorod arrays (NRAs) synthesized by the hydrothermal method. The corresponding reaction process can be expressed as:

(1) $$\mathrm{Ga}3++3\mathrm{OH}-\to \mathrm{Ga}\left(\mathrm{OH}\right)3$$

(2) $$\mathrm{Ga}\left(\mathrm{OH}\right)3\to \mathrm{GaOOH}+H2O$$

(3) Mg2++2GaOOH→α−Ga2O3−Mg+H2O

The scanning electron microscope (SEM) morphology of α‐Ga_2_O_3_ prepared directly from Ga(NO_3_)_3_ without Mg doping is characterized in Figure 1b. The obtained structure is a typical α‐Ga_2_O_3_ configuration consisting of rhombic nanorods with similar radial dimensions, clearly separated and uniformly distributed on the fluorine‐doped tin oxide (FTO) substrate. Research indicates that nanorod arrays offer significant benefits in PEC applications compared to bulk planar films.^[^ ^30^ ^]^ NRAs can act as antireflection layers, leading to excellent light capture efficiency. The geometry of nanowires results in the light absorption direction being orthogonal to the charge separation direction, which reduces the distance minority carriers must travel and enhances charge separation efficiency. Due to their larger surface area, they provide more active sites at the solid‐liquid interface for PEC reactions. The transmission electron microscope (TEM) diffraction pattern shown in Figure S1 (Supporting Information) demonstrates a nanorod lattice spacing of 0.251 nm, which aligns with the (110) crystal plane orientation of α‐Ga_2_O_3_. Elemental mapping by energy dispersive spectrometer (EDS) scanning illustrates a homogeneous distribution of Ga and O atoms within the nanorods, confirming the effective synthesis of α‐Ga_2_O_3_ nanorods. Based on analyses of different preparation conditions, the optimal parameter for the fabrication of 6% Mg‐doped α‐Ga_2_O_3_ was designated as Mg‐Ga_2_O_3_. Figure 1c illustrates the SEM morphology of the Mg‐Ga_2_O_3_ NRAs synthesized under the specific conditions described above. The image displays a significant morphological feature: rhombic nanorods have aggregated to form well‐defined structures. SEM images of Mg‐doped α‐Ga_2_O_3_ were captured under varying conditions of doping concentrations, temperatures, pH values, and growth times. These morphologies are depicted in Figures S2–S5 (Supporting Information). We found that the initial pH of the solution and the growth temperature during the hydrothermal process changed the nucleation form and rate of the crystals, and therefore had a greater effect on their morphology. The concentration and time of doping, on the other hand, mainly affect the aggregation state of the nanorods.

**Figure 2:**
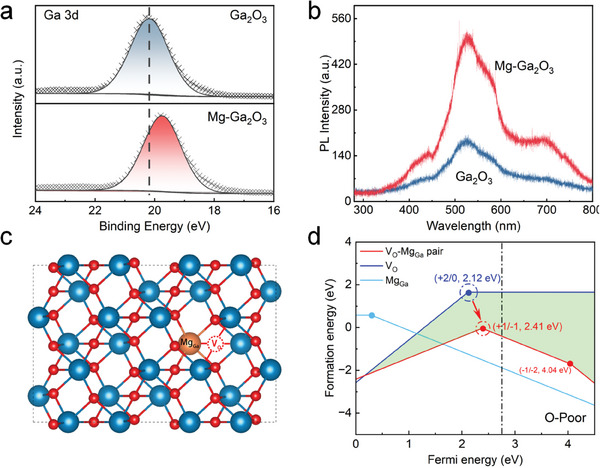
a) The XPS peak fitting of Ga *3d* core‐level spectrum. b) The PL spectra of α‐Ga_2_O_3_ and Mg‐Ga_2_O_3_. c) The conventional structure of α‐Ga_2_O_3_ and the different types of defects, d) The formation energies of defects in Mg‐doped α‐Ga_2_O_3_. The dashed line denotes the calculated bandgap of α‐Ga_2_O_3_.

**Figure 3:**
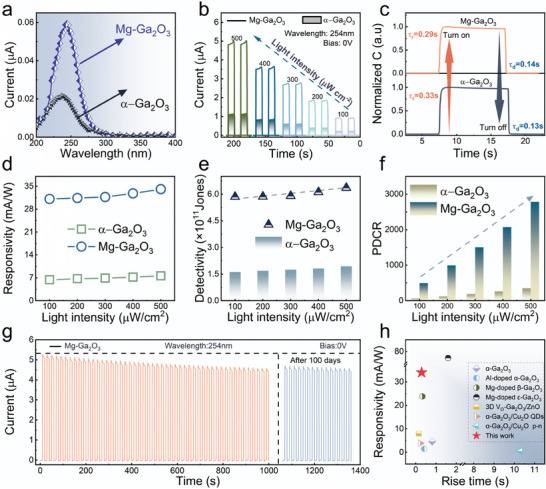
a) Spectral response of α‐Ga_2_O_3_ and Mg‐Ga_2_O_3_. b) Comparison of I‐T curves for two SBPDs at 0 V bias, 254 nm illumination, and varying light intensities. c) Time response comparison of the two SBPDs. d) The responsivity of the two SBPDs as a function of light power density. e) Detectivity comparison of the two SBPDs. f) Photo‐dark current ratio under varying light power densities. g) Stability test curve for the Mg‐Ga_2_O_3_ SBPD. h) Responsivity and time response of Mg‐Ga_2_O_3_ SBPD in comparison with previously reported works.

Figure 1d shows the TEM image of a single Mg‐Ga_2_O_3_ nanorod, while Figure 1e presents a high‐resolution image of the selected area. Fourier‐transform analysis (Figure 1f) reveals lattice spacings of 0.252 and 0.161 nm, which aligns with the (110) and (116) crystal plane orientations of α‐Ga_2_O_3_, respectively. EDS line scans are performed on the nanorods’ surface (Figure S6, Supporting Information), and elemental analysis of a single nanorod is demonstrated in Figure 1g (elemental composition is provided in Figure S7, Supporting Information), which characterized the content and distribution of various elements. Blue and green denotes the distribution of Ga and O within the individual nanorod. The images reveal adequate and homogeneous distributions of both Ga and O. The pink of Mg indicates the effective incorporation of Mg into the nanorods. These results confirm the successful incorporation of Mg into α‐Ga_2_O_3_ and the dopant is evenly distributed in α‐Ga_2_O_3_. The crystallographic properties of the synthesized samples were examined using X‐ray diffraction (XRD). Figure 1h shows that both samples exhibit a main peak at 36.01° and a faint feature peak at 64.71°, which aligns with the (110) and (116) planes of α‐Ga_2_O_3_ (JCPDS 43–1013). The above results align with TEM analysis and confirm the synthesized nanorods are α‐Ga_2_O_3_. The other peaks in the pattern correspond to characteristic peaks of the FTO substrate (SnO_2_), as indexed by JCPDS 46–1088. The peaks of the Mg‐Ga_2_O_3_ match the undoped sample, with no additional peak corresponding to impurities such as MgO or MgGaO, proving this preparation process can produce single‐phase Mg‐Ga_2_O_3_ NRAs. The XRD patterns of samples synthesized under other conditions are shown in Figures S8–S10 (Supporting Information) and the data indicates that no distinct (110) and (116) peaks were observed in Mg‐doped α‐Ga_2_O_3_ nanorods at 140 and 160 °C (C. SEM images also confirm that no distinct nanorod is detected at lower temperatures. Figure 1i displays the X‐ray photoelectron spectroscopy (XPS) spectra for both undoped and doped samples after etching. Figure 1i‐1 shows the Ga 2*p*3/2 and Ga 2*p*1/2 peak splitting. Due to the lower electronegativity of Mg compared to Ga,^[^ ^31^, ^32^, ^33^ ^]^ the Mg─O bond is more readily formed than the Ga─O bond. Consequently, the peak positions of Ga elements shift to lower binding energies. The O 1*s* core‐level spectrum in Figure 1i‐2 confirms the formation of Mg‐O bonds, and the detection of the Mg 1*s* core‐level (Figure 1i‐3) provides further evidence for the successful incorporation of Mg into the Ga_2_O_3_ nanorods.

Doping significantly impacts intrinsic defects and defect complexes in semiconductor materials. The following analysis, based on experimental characterization techniques and first‐principles calculations, examines the effect of Mg doping on intrinsic defects and/or defect complexes in α‐Ga_2_O_3_ nanorod arrays. **Figure** **2**a shows the Ga 3*d* core‐level spectrum. After the incorporation of Mg, a shift in the Ga 3*d* binding energy peak from 20.17 to 19.73 eV was observed, indirectly validating a significant increase in the concentration of V_O_.^[^ ^34^ ^]^ Figure 2b depicts the photoluminescence (PL) spectroscopy analysis of two samples, providing crucial insights into the V_O_ characteristics.^[^ ^35^, ^36^ ^]^ Under the excitation of a room‐temperature continuous laser at a wavelength of 325 nm, both samples exhibit emission in the 400–750 nm wavelength range. As is well known, the emission of Ga_2_O_3_ in the visible light range is closely associated with V_O_.^[^ ^37^, ^38^, ^39^ ^]^ Figure 2b shows that Mg doping induces stronger emission in the visible range, suggesting that Mg doping leads to an increase in V_O_, which is consistent with the XPS analysis. To better understand the interaction between Mg impurities and V_O_ defects, a detailed investigation is conducted using defect formation energy and ionization energy. Generally, the formation energy H^*f*^(D^*q*^, α − *Ga*_2_ *O*_3_) for a dopant D at site A in a charged state q, denoted as D^q^, can be expressed as:

(4) $$\begin{array}{ccc} H^{f}\left(D^{q},\alpha -Ga_{2}O_{3}\right) & = & E_{t}\left(D^{q},\alpha -Ga_{2}O_{3}\right)-E_{t}\left(\alpha -Ga_{2}O_{3}\right)\\ & & +\sum \Delta n_{i}\mu_{i}+q\left(E_{VBM}+E_{f}+\Delta V\right)+\Delta E^{q} \end{array}$$

E_*t*_(D^*q*^, α − *Ga*_2_ *O*_3_) and E_*t*_(α − *Ga*_2_ *O*_3_) represent the total energies of the supercell with and without the dopant, respectively.^[^ ^40^, ^41^, ^42^, ^43^ ^]^ Δn_*i*_ denotes the number of atoms of Ga, Mg, or O removed (+) from or added (‐) to the ideal supercell, while µ_i_ refers to the chemical potential of the constituent atoms of type i. E_VBM_ indicates the valence band maximum (VBM), and E_f_ is the Fermi level, which is associated with the charged states. The correction term ΔV represents the average potential difference between the doped supercell and the undoped one. ΔE^q^ accounts for the correction of the spurious *Coulomb* interactions of charged defects arising from the use of periodic boundary conditions. Since the α‐Ga_2_O_3_ was subjected to vacuum annealing during preparation, the oxygen environment was considered oxygen‐poor. The chemical potential µ_Ga_ is referenced to the total energy per atom of bulk Ga, while µ_O_ is referenced to the energy of an oxygen atom in the stable phase of α‐Ga_2_O_3_.^[^ ^44^, ^45^, ^46^ ^]^ The computational model is shown in Figure 2c.^[^ ^47^, ^48^ ^]^ Figure 2d illustrates the defect formation energy and ionization energy. It is evident that the formation energy of V_O_‐Mg_Ga_ is significantly lower than that of V_O_ alone. This phenomenon is similar to the donor–acceptor co‐doping strategy, indicating that Mg incorporation effectively enhances the V_O_ concentration and readily promotes the formation of V_O_‐Mg_Ga_ complex defects.^[^ ^49^ ^]^ Furthermore, the ionization energy of donor‐like V_O_‐Mg_Ga_ complex defects is lower than that of isolated V_O_, confirming that these complexes more effectively contribute carriers.^[^ ^50^, ^51^ ^]^ Both experimental and theoretical results demonstrate that the low‐cost hydrothermal synthesis of α‐Ga_2_O_3_ nanorod arrays, combined with Mg doping, induces the formation of V_O_‐Mg_Ga_ complexes. These complexes not only increase the V_O_ concentration but also elevate the donor energy level of V_O_, which is expected to have a significant impact on device performance.

To extend Ga_2_O_3_‐based photodetectors for underwater applications, this study proposes the construction of Mg‐doped α‐Ga_2_O_3_ SBPD based on the PEC. Figure S11 (Supporting Information) depicts the preparation process flow and an image of the photodetector. **Figure** **3**a illustrates the spectral response range of α‐Ga_2_O_3_ and Mg‐Ga_2_O_3_ SBPDs at 0 V bias, both exhibiting peak responses at 240 nm and a cutoff wavelength ≈280 nm. Figure S12 (Supporting Information) presents the detectors’ response to 365 nm UV light, showing that neither the undoped nor the doped detectors exhibit a detectable response. This suggests that both α‐Ga_2_O_3_ and Mg‐Ga_2_O_3_ SBPDs possess favorable solar‐blind characteristics capable of detecting UVC signals. Importantly, the Mg‐Ga_2_O_3_ SBPD demonstrates superior photocurrent response under identical illumination conditions. Further experiments were conducted to assess the detectors’ response to 254 nm radiation, varying the light intensity from 100 to 500 µW cm^−2^ at 0 V bias. Figure 3b presents the I‐T data and the photocurrent response of α‐Ga_2_O_3_ and Mg‐Ga_2_O_3_ SBPDs. It can be clearly seen that the photocurrent increases with increasing power density for both detectors. The photocurrent of the Mg‐Ga_2_O_3_ SBPD (4.96 µA) was ≈4.35 times greater than that of α‐Ga_2_O_3_ SBPD (1.14 µA) at 500 µW cm^−2^. Figure S13 (Supporting Information) is the I‐T curves of the Mg‐Ga_2_O_3_ SBPD under different bias voltages and the light intensity of 400 µW cm^−2^. Figure S14 (Supporting Information) depicts I‐V curves at different light intensities, which further confirms the device's self‐powered feature.

In practical applications of a photodetector, the key indexes include response time (t), responsivity (R), detectivity (D), and the photo‐dark current ratio (PDCR). Figure 3c displays the normalized rise and decay times of the photocurrent for two samples. The rise time is defined as the duration required for the photocurrent to increase from 10% to 90% of its peak value, while the decay time is the interval for the photocurrent to decrease from 90% to 10%. The data indicates that while the Mg‐Ga_2_O_3_ SBPD enhances photocurrent significantly, its response times (t_r_/t_d_ = 0.29/0.14 s) are comparable to those of the α‐Ga_2_O_3_ (t_r_/t_d_ = 0.33/0.13 s). The responsivity of the detector was calculated using the ratio of output current to input radiation signal power:^[^ ^52^ ^]^

(5) $$R_{\lambda }=\frac{I_{p}-I_{d}}{PS}$$

where *I_p_* represents the photoresponse current, *I_d_* denotes the dark current, *P* signifies the incident optical power, and *S* indicates the photoreceptor area. Figure 3d displays the specific responsivity of two photodetectors at a wavelength of 254 nm and varying optical powers without external bias. The results demonstrate that the responsivities of the photodetectors increase with the rise in incident light intensity. At 500 µW cm^−2^, two photodetectors exhibit their highest responsivities of 7.69 and 34.54 mA W^−1^ for α‐Ga_2_O_3_ and Mg‐Ga_2_O_3_ SBPD, respectively. The responsivity improved by more than 400%. We then proceed to evaluate the specific detection rate, which quantifies the detector's capability to identify the minimum detectable signal. A higher value indicates greater sensitivity to weak solar‐blind light. It can be calculated using the equation:^[^ ^53^ ^]^

(6) $$D^{*}=\frac{R\sqrt{S}}{\sqrt{2eI_{d}}}$$

where *e* signifies the elementary charge. Figure 3e shows that the maximum detectivities of α‐Ga_2_O_3_ and Mg‐Ga_2_O_3_ SBPDs are 1.94 × 10¹¹ and 6.36 × 10¹¹ *Jones*, respectively. The PDCR of a device quantifies the disparity between the output signal and background noise. A higher signal‐to‐noise ratio results in a greater sensitivity to solar‐blind signals. It could be computed by the formula:^[^ ^54^ ^]^

(7) $$PDCR=\frac{I_{p}-I_{d}}{I_{d}}$$

Figure 3f presents the *PDCR* values for both samples under consistent conditions. The calculated maximum *PDCR* for α‐Ga_2_O_3_ and Mg‐Ga_2_O_3_ SBPDs are 3.75 × 10^2^ and 2.74 × 10^3^ under 500 µW cm^−2^, respectively. Figures S15–S18 (Supporting Information) illustrate the performance characteristics of PEC photodetectors fabricated with Mg‐doped α‐Ga_2_O_3_ nanorods at various doping concentrations and growth conditions.

Stability is also an essential criterion for evaluating the practical value of a detector. As depicted in Figure 3g, a Mg‐Ga_2_O_3_ SBPD underwent 1000 s cyclic test followed by a retest after exposure to air for 100 days. The results clarify that the current of the device decreased slightly during the 1000 s cyclic test, which may be due to the unstable test environment in the initial phase. However, after being exposed for 100 days, the detector's photocurrent remains essentially unchanged compared to its initial value. This underscores the excellent stability of the fabricated Mg‐Ga_2_O_3_ SBPD. As shown in Figure 3h (more details are provided in Table S2, Supporting Information), the Mg‐Ga_2_O_3_ obtained in this work can output response rates of 34.54 mA W^−1^ and a response time of 0.29/0.14 s under 254 nm UVC light of 500 µW cm^−2^. A brief review of recent studies indicates that Mg‐Ga_2_O_3_ has demonstrated significant improvements across various metrics compared to pristine structures. These advancements prove the substantial potential of Mg‐doped α‐Ga_2_O_3_ for applications in solar‐blind detection.

**Figure 4:**
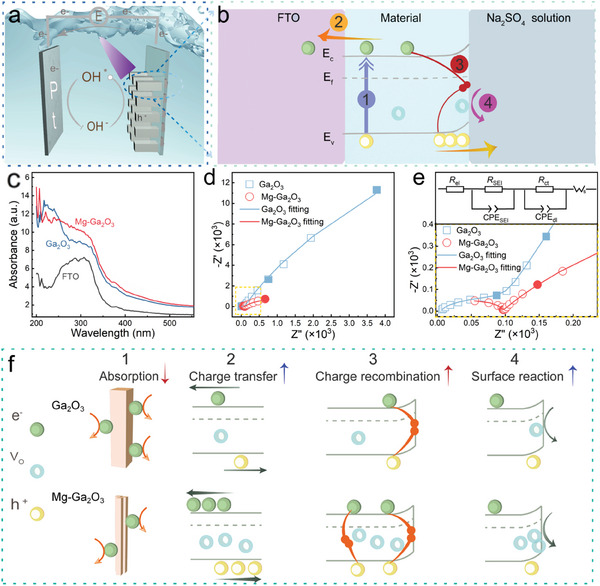
a) The schematic diagram of the operating principle of the PEC device. b) The interface of the PEC photodetector fabricated in this work. c) UV–vis absorption spectra of α‐Ga_2_O_3_ and Mg‐Ga_2_O_3_ SBPDs. d) Electrochemical impedance spectra (EIS) for α‐Ga_2_O_3_ and Mg‐Ga_2_O_3_ SBPDs. e) The equivalent current and corresponding magnified plots. f) The mechanism diagram of α‐Ga_2_O_3_ and Mg‐Ga_2_O_3_ SBPDs.

In this PEC system, each component contributes independently and collaborates with the formed interfaces. The principles are displayed in **Figure** **4**a,b. Electron–hole pairs are generated when the energy of the incident photons exceeds the bandgap of the material. When a semiconductor contacts with a liquid electrolyte, the electrostatic potential difference at the semiconductor‐electrolyte interface leads to changes in the band structure. Due to the presence of the intrinsic electric field and the high mobility of charge carriers, the photo‐excited carriers undergo spatial separation. Negative charged carriers traverse through the semiconductor reach the counter electrode and participate in a reduction half‐reaction. Meanwhile, holes accumulate in the upward‐bent energy band, where they react with hydroxide ions (OH^−^) and complete the circuit through an oxidation half‐reaction.^[^ ^55^, ^56^ ^]^ The detailed mechanism at the solid‐electrolyte interface requires further discussion. When referring to the mechanism of the solid‐electrolyte interface in Mg‐doped α‐Ga_2_O_3_, four competitive factors should be considered to assess their impacts: light absorption efficiency, charge recombination, charge transfer, and the electrocatalytic reactions occurring at the Helmholtz layer of the solid‐electrolyte interface. After Mg doping, the absorption of FTO, α‐Ga_2_O_3_, and Mg‐Ga_2_O_3_ in UV–vis range are provided in Figures 4c and S19 (Supporting Information). It shows that the absorption peak of FTO is ≈300 nm. The absorptions of α‐Ga_2_O_3_ and Mg‐Ga_2_O_3_ SBPDs are primarily in the solar‐blind region. In comparison to α‐Ga_2_O_3_, Mg‐Ga_2_O_3_ exhibits a lower efficiency of light absorption efficiency. This decrease might be due to the creation of aggregated nanorods, leading to a reduction in the specific surface area.

To investigate the charge transfer resistance and the influence of the incorporation of Mg ions, Figure 4d illustrates the EIS of α‐Ga_2_O_3_ and Mg‐Ga_2_O_3_ SBPDs. The impedance spectra reveal high‐/medium‐frequency semicircles, along with low‐frequency arcs. These findings align with earlier results reported by Opra et al.,^[^ ^42^ ^]^ who investigated Zr‐doped anatase TiO_2_ using EIS. They considered that the semicircles correspond to electron transport through the FTO interfacial layer (SEI) and charge migration in the solid‐liquid double layer.^[^ ^57^ ^]^ The low‐frequency arcs are related to ionic diffusion resistance.^[^ ^58^ ^]^ Notably, the mid‐frequency semicircle diameters in the doped structure are significantly smaller than those in the undoped one, implying a substantial enhancement in conductivity via Mg doping. The yellow part of the EIS graph is magnified in the lower half of Figure 4e. By fitting the EIS data, an equivalent circuit in the upper half of Figure 4e was constructed, in which a constant phase element is applied to account for material inhomogeneities in place of a simple capacitor. The equivalent circuit includes the ohmic resistance of the electrolyte (R_el_), the SEI resistance (R_SEI_) coupled with the layer capacitance (CPE_SEI_), the charge transfer resistance (R_ct_) coupled with the double‐layer capacitance (CPE_dl_), and the Warburg impedance (W_s_), which is related to ionic diffusion dynamics. From the Nyquist plots and fittings, the values of R_el_ and R_SEI_ for α‐Ga_2_O_3_ and Mg‐Ga_2_O_3_ are virtually equal. The charge transfer R_ct_ sharply decreased from 89.09 KΩ in α‐Ga_2_O_3_ to 13.71 KΩ in Mg‐Ga_2_O_3_, proving the incorporation of Mg could markedly lower the charge transfer resistance. This observation further confirms that Mg doping induces the formation of V_O_‐Mg_Ga_ complexes, which effectively lower the ionization energy of V_O_. Consequently, this enhances the material's conductivity and promotes carrier transport, thereby benefiting the performance of the device.^[^ ^59^, ^60^ ^]^

Figure 4f illustrates, through a schematic mechanism diagram, the delicate balance among four competing factors in the PEC process. Morphological changes did not significantly enhance the photoanode's light absorption efficiency, thus failing to improve device performance. In contrast, the increase in V_O_ concentration is closely tied to the other three factors, playing a critical role in the PEC process. An appropriate concentration of V_O_ can modulate the electronic structure of Ga_2_O_3_, promoting the formation of V_O_‐Mg_Ga_ complexes. This adjustment enhances the material's electron concentration, reduces interfacial resistance, and shortens charge retention time, significantly improving charge migration efficiency. Moreover, an increased V_O_ concentration can lower interfacial barriers, reducing energy losses during charge transfer and further boosting charge separation and transfer efficiency.^[^ ^61^ ^]^ Additionally, V_O_ acts as new active sites, serving as catalytic centers that more effectively adsorb and convert reactants, facilitating interfacial oxidation half‐reactions and enhancing PEC performance. However, an excessive V_O_ concentration can lead to interfacial hole accumulation, exacerbating surface recombination or penetrating into the material's bulk, intensifying bulk recombination effects. This shortens carrier lifetimes, negatively impacting PEC response time and current generation.^[^ ^61^ ^]^ Therefore, the influence of Mg doping‐induced V_O_ defects on the photoelectric performance of α‐Ga_2_O_3_ nanorods is primarily reflected in the competition between charge transfer, interfacial catalysis, and charge recombination. Despite the complexity of these factors, experiments have demonstrated that appropriate Mg doping induces optimal V_O_ defects, which favorably enhance the UVC detection capability of α‐Ga_2_O_3_ nanorod arrays.

**Figure 5:**
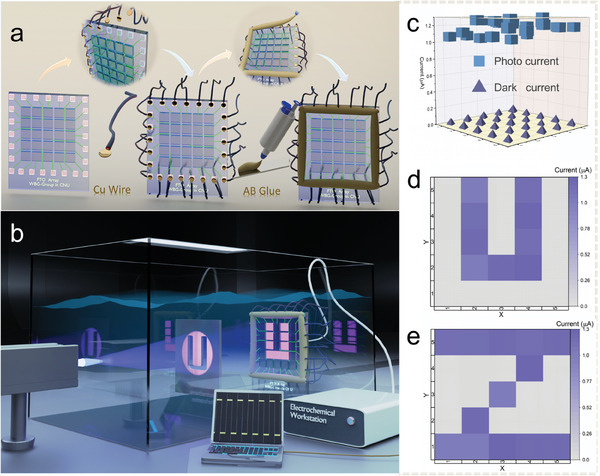
a) The preparation process of the 5 × 5 matrix array. b) The underwater imaging system. c) Average photocurrent under illumination at 500 µW cm^−2^ (254 nm) and dark current curve for the Mg‐Ga_2_O_3_ 5 × 5 array. d,e) 2D thermal images of “U” and “Z”, respectively.

Underwater imaging is of great importance, and the advantage of PEC photodetectors over other solid‐state devices lies in their capability to operate effectively in moist environments. Therefore, an experimental design was formulated for a 5 × 5 PEC solar‐blind imaging array utilizing Mg‐Ga_2_O_3_, aiming to fabricate a bias‐free underwater imaging system. **Figure** **5**a depicts the schematic illustration of a 5 × 5 matrix array of PEC‐type Mg‐Ga_2_O_3_. Each Mg‐Ga_2_O_3_ pixel has an effective area of 3 × 3 mm^2^. The imaging unit uses 25 copper wires with conductive silver adhesive to connect the electrodes and is wrapped in commercial AB epoxy and then cured in a vacuum. Figure 5b illustrates the concept of solar‐blind imaging utilizing Mg‐Ga_2_O_3_‐based devices. A 5 × 5 matrix is positioned beneath a hollow mask etched “U” at 500 µW cm^−2^ UV light. The remaining detection locations are either retained in darkness or exposed to feeble UV light that penetrates the mask to reach the Mg‐doped α‐Ga_2_O_3_ imaging array. This section uses a two‐electrode test system based on practical requirements. The mask engraved with the “U” pattern functions as the counter electrode, while the imaging array serves as the working electrode. Under external solar‐blind UV radiation, light penetrates through the mask and induces charge separation/transfer at the respective imaging components. This electrical signal is detected by an electrochemical workstation and the current from each individual imaging unit is displayed on the receiving computer.^[^ ^62^ ^]^ The homogeneity of the arrays is a fundamental requirement for integrated applications and must be studied before solar‐blind imaging. Figure 5c displays the average photocurrent density of each cell within the Mg‐Ga_2_O_3_ array under uniform light intensity. The results indicate that the photocurrent densities for all units fall within the same order of magnitude under identical light intensity, demonstrating remarkable uniformity. Figure 5d,e depict 2D maps of the Mg‐Ga_2_O_3_ PEC imaging system showcasing the letters “U” and “Z”. It is noteworthy that the photocurrent response of each imaging unit under 254 nm light intensity is highly uniform, and the minimal dark current has negligible impact on underwater photography during actual testing. This demonstrates that the uniformity and resolution of each individual photoelectrode within the device are sufficiently practical, laying the groundwork for future applications of PEC SBPDs in underwater imaging systems.

## 3 Conclusion

This study successfully developed a self‐powered photoelectrochemical solar‐blind photodetector based on Mg‐doped α‐Ga_2_O_3_ nanorod arrays, demonstrating excellent performance for underwater imaging. Using a cost‐effective hydrothermal synthesis method, Mg doping was introduced, significantly altering the morphology and properties of α‐Ga₂O₃. DFT calculations confirmed that Mg atoms primarily substitute Ga sites, forming V_O_‐Mg_Ga_ complexes with high doping efficiency. These defects enhance oxygen vacancy concentration, interfacial catalytic activity, and carrier separation and transfer efficiency during the PEC process. The Mg‐doped α‐Ga_2_O_3_ PEC‐SBPD exhibited a responsivity of 34.54 mA W^−1^ and a detectivity of 6.36 × 10^11^ Jones under 254 nm UV illumination at zero bias, with a 435% improvement in photocurrent response compared to undoped α‐Ga_2_O_3_. Furthermore, the successful integration of a 5 × 5 PEC‐SBPD array enabled high‐resolution solar‐blind imaging in underwater environments, demonstrating excellent uniformity and practicality. This work highlights the potential of Mg‐doped α‐Ga_2_O_3_ in solar‐blind detection and underwater imaging, providing a robust foundation for future advancements in photoelectrochemical photodetectors for extreme environmental applications.

## 4 Experimental Section

### Reagents and Substrate

In this experiment, five chemical reagents were employed: Mg and Ga sources were derived from magnesium sulfate heptahydrate (MgSO_4_·7H_2_O) and gallium nitrate nonahydrate (Ga(NO_3_)_3_·9H_2_O), respectively. pH adjustment was achieved using sodium hydroxide (NaOH) and dilute sulfuric acid (H_2_SO_4_). The PEC test solution was sodium sulfate (Na_2_SO_4_), all of the above were of analytical grade. The transparent FTO glass with a resistance of 7 Ω and dimension of 1 × 2 cm served as the substrate for material growth. Deionized water was sourced from the laboratory.

### Preparation and Modulation of Materials

The synthesis of Mg‐doped α‐Ga_2_O_3_ nanorods was conducted using a facile hydrothermal synthesis technique, as illustrated in Figure S20 (Supporting Information). α‐Ga_2_O_3_ nanorod arrays were synthesized by dissolving 4 g of Ga(NO_3_)_3_·9H_2_O in 40 mL of deionized water and agitating the solution with a magnetic stirrer for 10 min at room temperature. The stirred precursor solution was adjusted to pH 4 using 0.15 g mL^−1^ of NaOH solution and then transferred to a 100 mL Teflon container holding an FTO substrate, which was then placed in a hydrothermal reactor for material growth. Different doping concentrations were achieved by varying the chemical composition of the precursor solutions: 4 g of Ga(NO_3_)_3_·9H_2_O and different amounts of MgSO_4_·7H_2_O (0.12, 0.23, 0.24, 0.31, and 0.40 g) dissolved in 40 mL of deionized water, during this the remaining growth conditions remain unchanged. When the doping concentration was determined to be 6%, the pH was regulated by the varied diluted sulfuric acid (pH values: 1, 1.5, 2, 3.5, and 4) to explore the effect of different pH values while keeping other growth parameters unchanged. Subsequently, the doping concentration of Mg was determined to be 6%, and the initial solution with Ph = 2 was used to further explore the optimal temperature for material growth. After conducting temperature studies with 20 °C as a gradient, 180 °C was found to be the optimal value for growth. The optimum growth time for the nanorods was finally determined when the Mg ion doping was 6%, the pH was 2, and the growth temperature was 180 °C. Five different growth times of 4, 8, 12, 18, and 24 h were tested. It was found that the performance of Mg‐doped α‐Ga_2_O_3_ nanorods grown at 18 h was the best (refer to Table S1, Supporting Information for a summary of the data).

### Material Characterization and Performance Tests

The microscopic morphology and elemental composition were obtained by SEM (Thermo Scientific Apreo 2C) and HR‐TEM (Talos F200S G2), the chemical bonding states were analyzed using XPS (Thermo Fischer, ESCALAB Xi+, USA), the structure was identified by XRD (XRD, Bruker D8 ADVANCE A25X), the absorption spectrum was tested using a UV–vis spectrophotometer (Hitachi, U‐4100).

### Device Preparation and Application Design

Electrical characterization was performed on an electrochemical workstation (Chenhua Instrument Co., Ltd.) using a three‐electrode configuration. The nanomaterials grown on FTO substrates served as the working electrode, a platinum foil (10 × 15 mm^2^) as the counter electrode, and a saturated calomel electrode (SCE) as the reference electrode. A UVLS‐28 EL UV lamp provided UV light at wavelengths of 254 and 365 nm. The electrolyte was a 0.5 m Na_2_SO_4_ aqueous solution. The illuminated area was maintained at 0.28 cm^2^. EIS measurements were conducted at a light intensity of 700 µW cm^−2^ and 1.0 kHz. To enable underwater imaging applications, the following experimental setup was designed: an array of photovoltaic electrodes composed of Mg‐doped α‐Ga_2_O_3_ nanorods was grown on patterned 5 × 5 arrays of FTO substrates (25 × 30 × 1.1 mm) using the synthesis method described above, with the photovoltaic pixel in the imaging array has an effective area of ≈3 × 3 mm^2^.

## Conflict of Interest

The authors declare no conflict of interest.

## Supporting information



Supporting Information

## Data Availability

The data that support the findings of this study are available in the supplementary material of this article.
