# Supplementary material for: Mg‐doped α‐Ga2O3 Nanorods for the Construction of Photoelectrochemical‐Type Self‐Powered Solar Blind UV Photodetectors and Underwater Imaging Application
Source: Adv Sci (Weinh). 2025 Feb 26;12(16):2413074. doi: 10.1002/advs.202413074 (PMC12021088; doi:10.1002/advs.202413074)
Supplement: Supplementary file 1 — Supporting Information [file ADVS-12-2413074-s001.docx]

*Supporting Information*

**Mg-doped α-Ga_2_O_3_ nanorods for the construction of photoelectrochemical-type self-powered solar blind UV photodetectors and underwater imaging application**

*Xin Zhou^#ab^, Lijuan Ye^#b^, Lai Yuan^b^, Dan Zhang^b^, Hong Zhang ^b^, Di Pang ^b^, Yan Tang ^b^, Honglin Li^*abd^, Wanjun Li^*b^, Heping Zeng^*acd^*

X. Zhou, L Ye, L. Yuan, D. Zhang, H. Zhang, D. Pang, Y. Tang, H. Li, W. Li, H. Zeng

^a^Chongqing Institute for Brain and Intelligence, Guangyang Bay Laboratory, Chongqing, 400064, China

^b^College of Physics and Electronic Engineering, Chongqing Normal University, Chongqing, 401331, China

^c^State Key Laboratory of Precision Spectroscopy, East China Normal University, Shanghai 200241, China

^d^Chongqing Key Laboratory of Precision Optics, Chongqing Institute of East China Normal University, Chongqing 401120, China

*# These authors contributed equally to the work.*

E-mail: *lin@cqnu.edu.cn (H. Li), liwj@cqnu.edu.cn (W. Li),hpzeng@phy.ecnu.edu.cn (H. Zeng)*

**
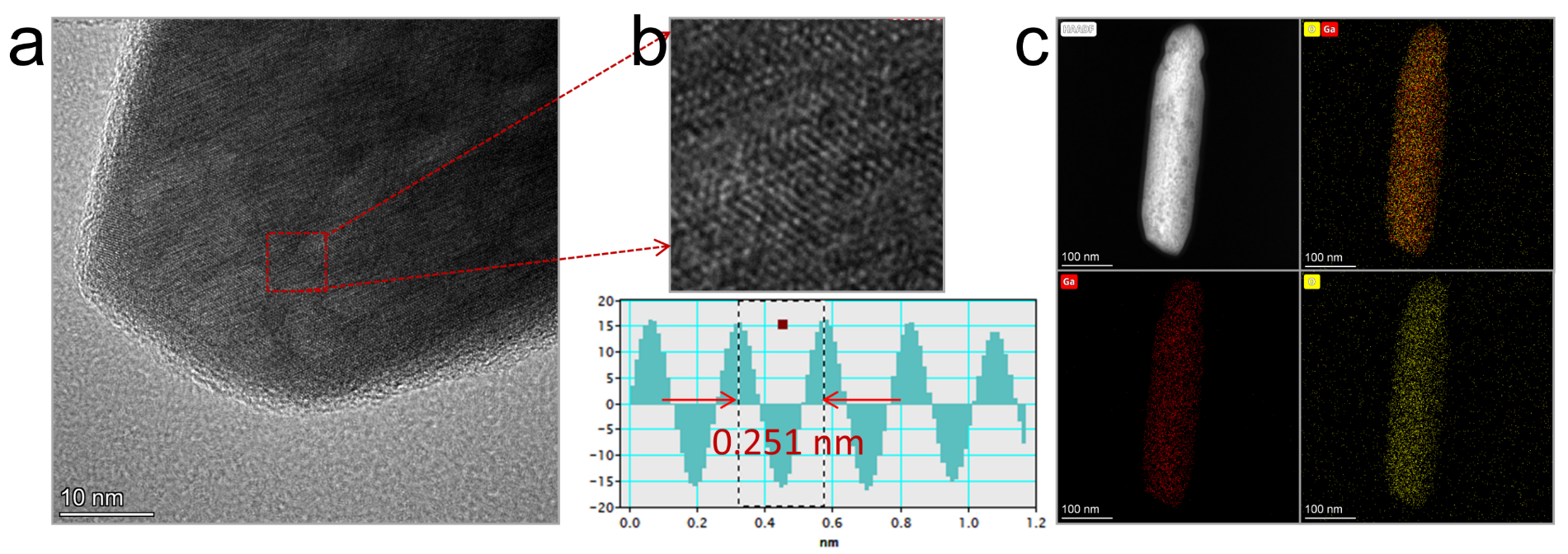
Figure S1** TEM image of α-Ga_2_O_3_ nanorod and the corresponding lattice spacing.


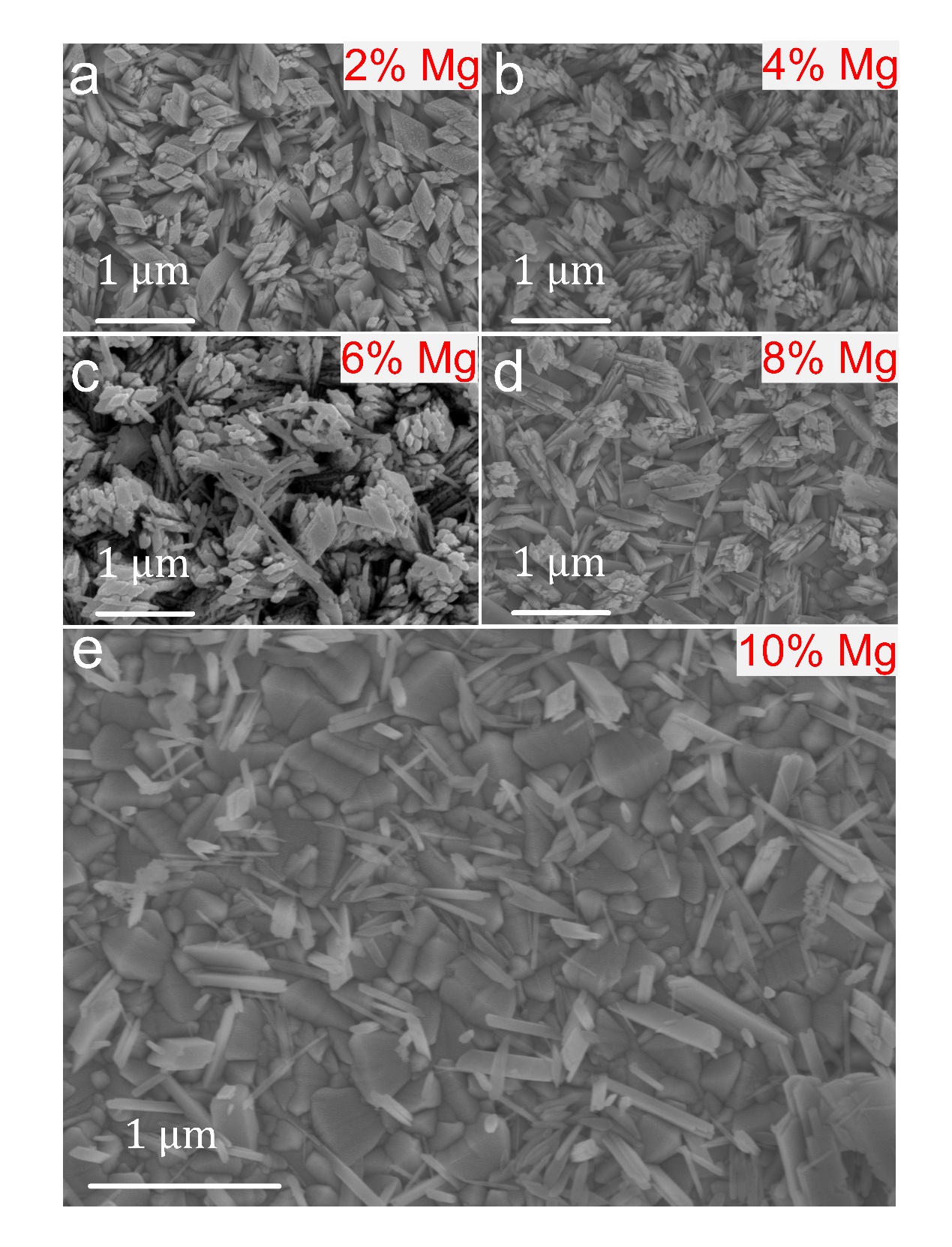
**Figure S2** SEM images of Mg-doped α-Ga_2_O_3_ at different Mg doping concentrations.


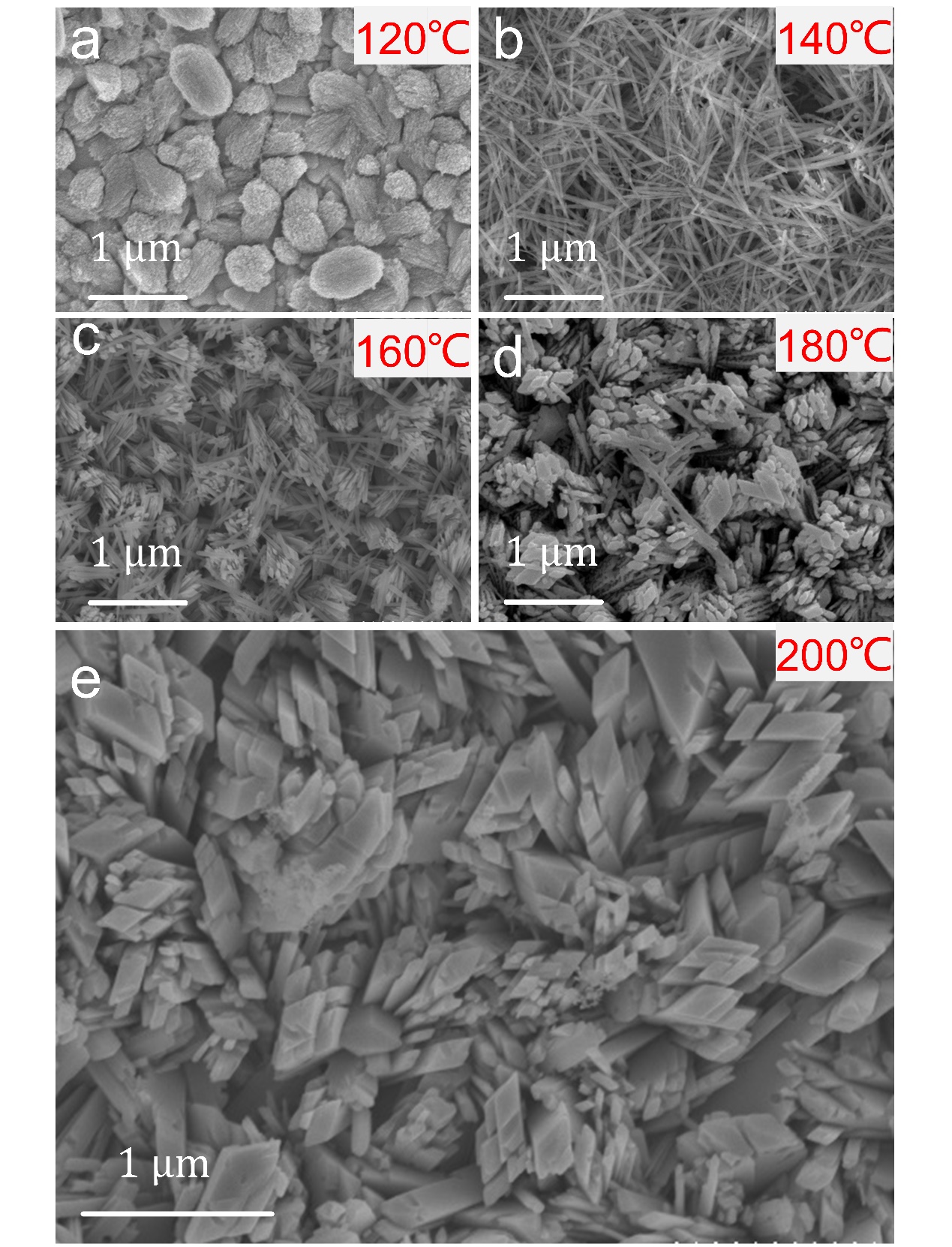


**Figure S3** SEM images of Mg-doped α-Ga_2_O_3_ at different temperatures.


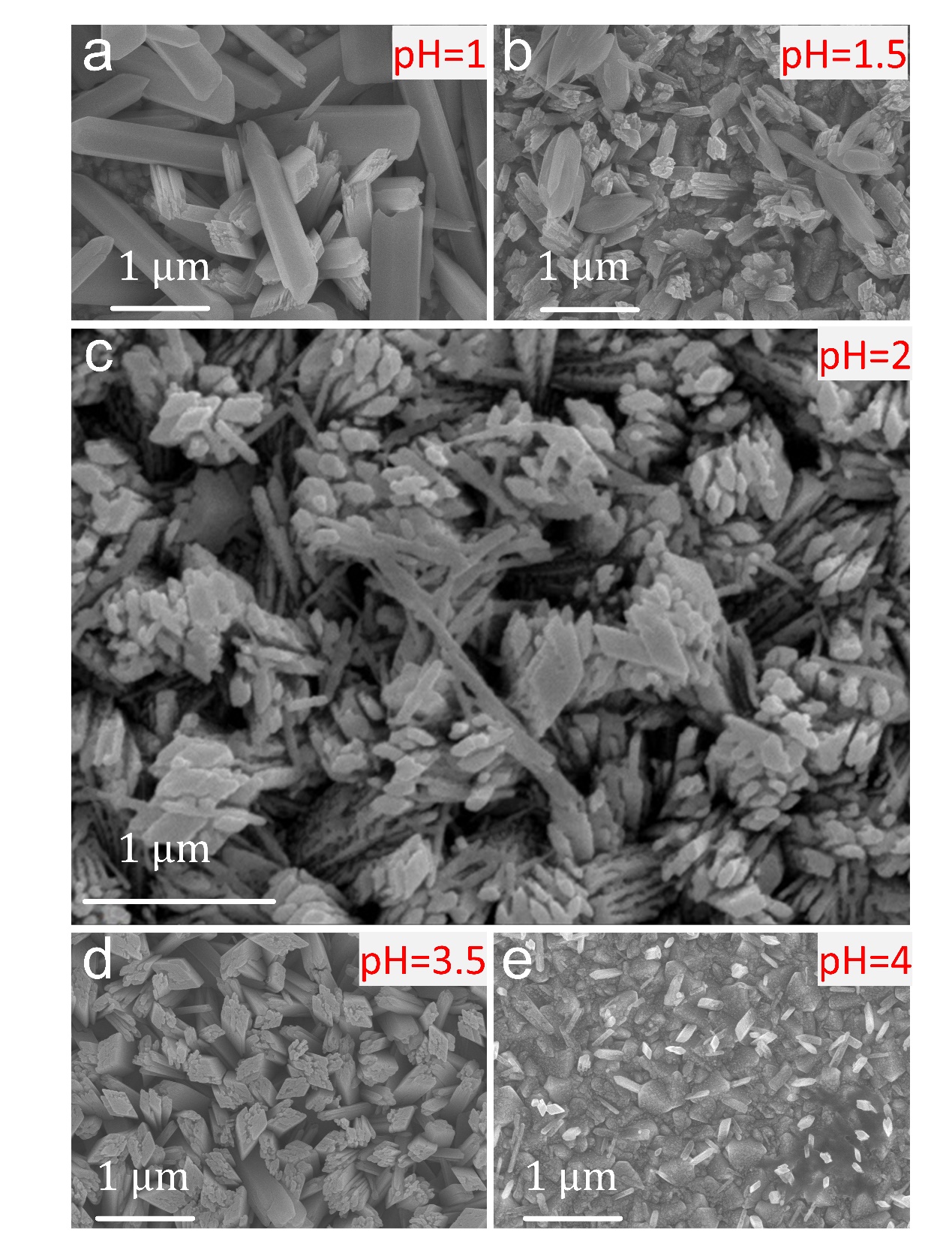


**Figure S4** SEM images of Mg-doped α-Ga_2_O_3_ at different pH values.


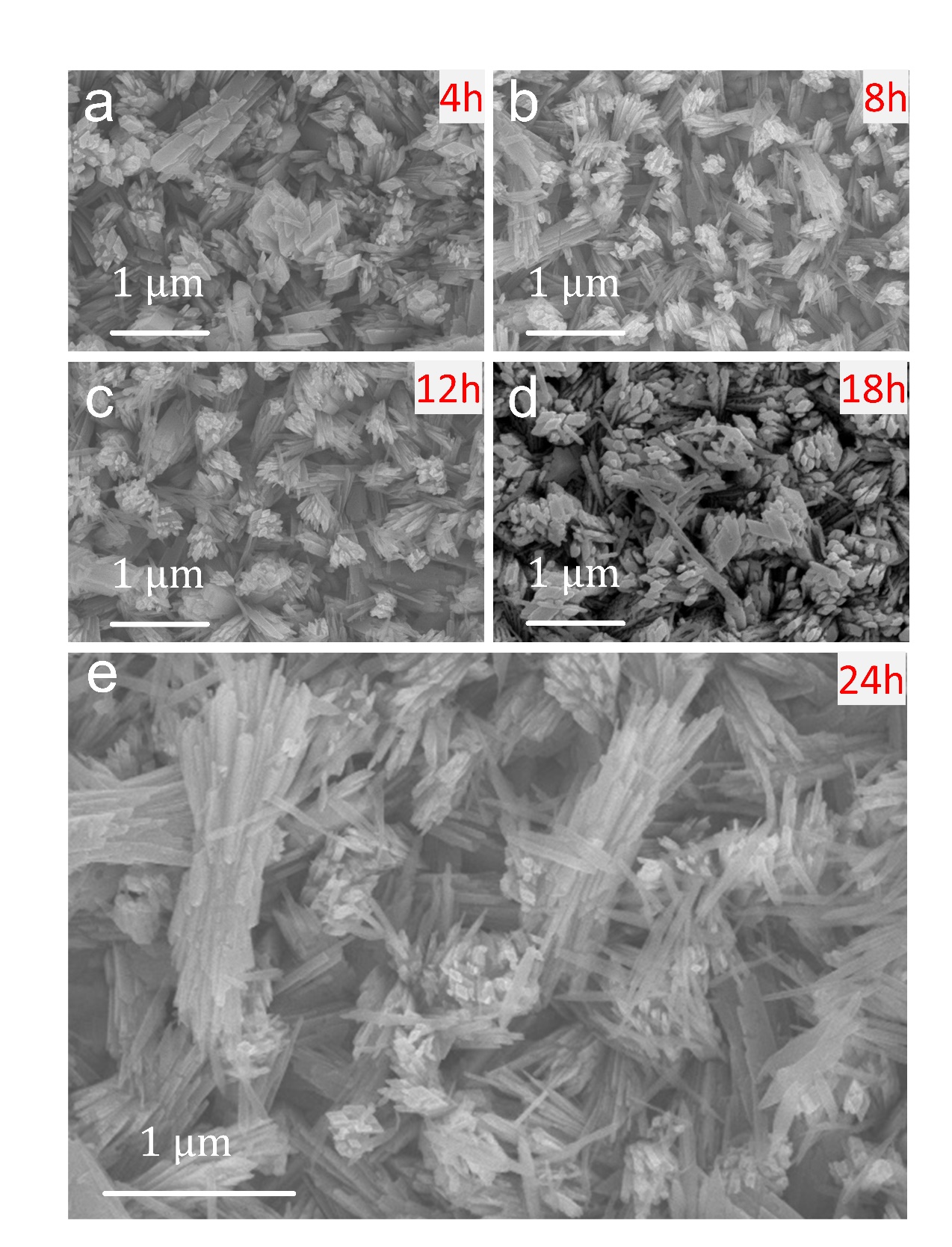
**Figure S5** SEM images of Mg-doped α-Ga_2_O_3_ at different reaction times.


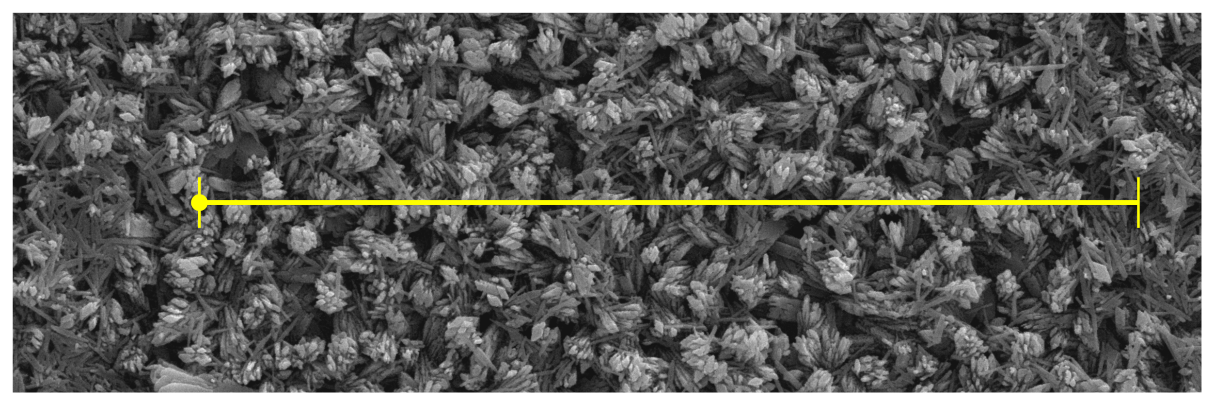


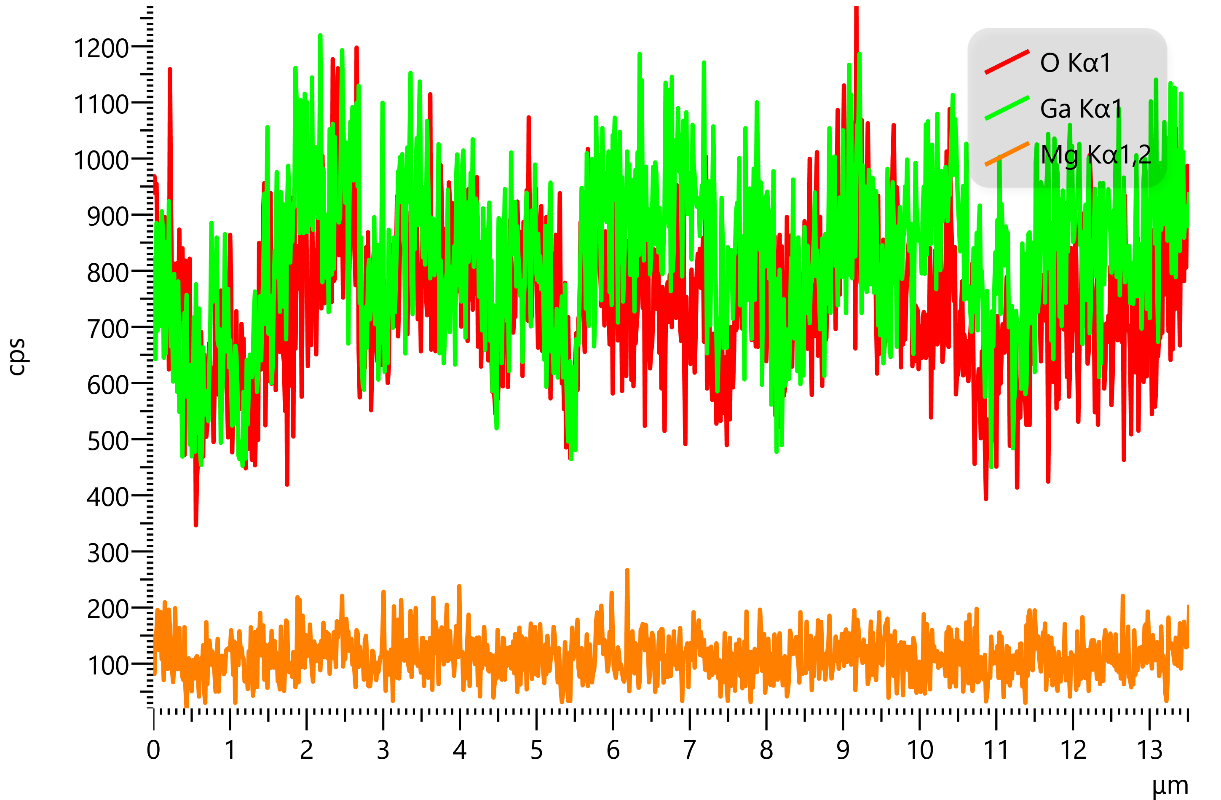


**Figure S6** Line-scan elemental analysis of Mg-doped α-Ga_2_O_3_.


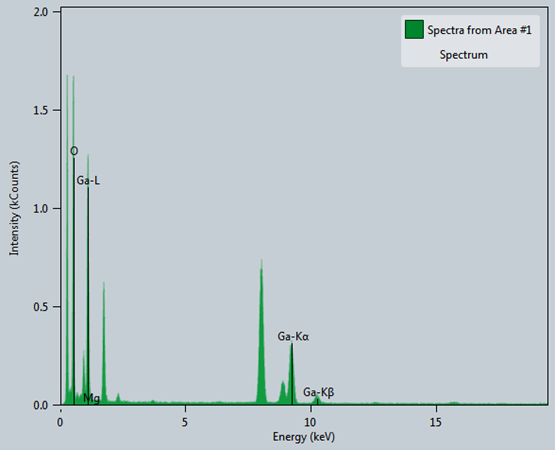


**Figure S7** TEM EDS elemental spectrum of Mg-doped α-Ga_2_O_3_.


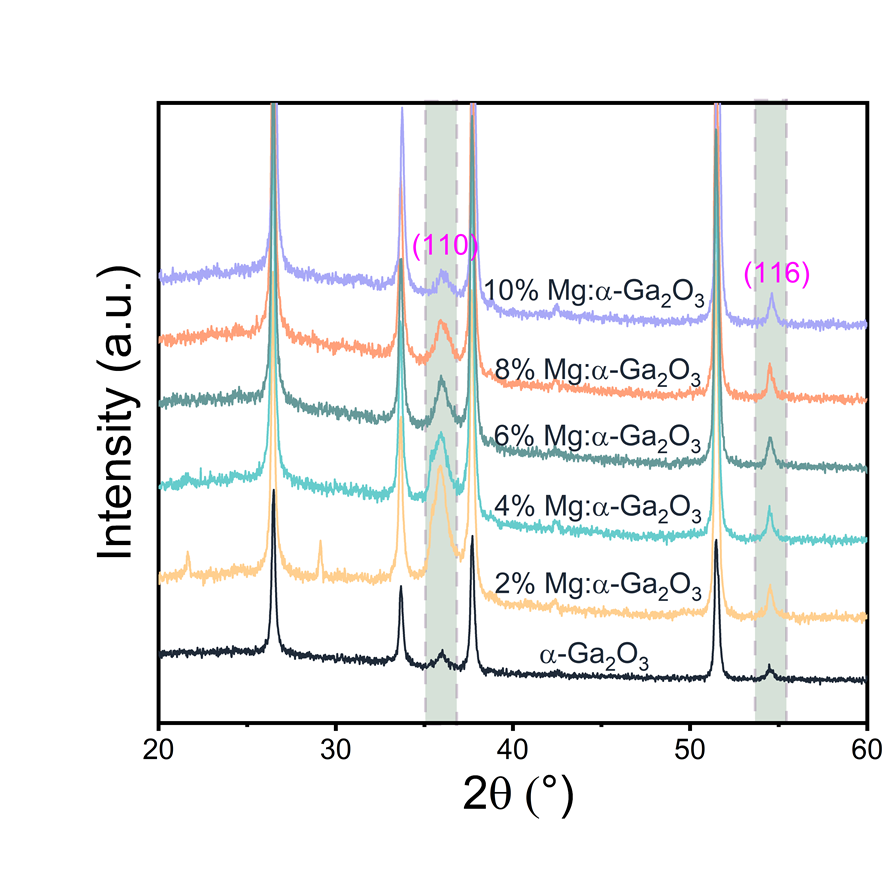
**Figure S8** XRD patterns of Mg-doped α-Ga_2_O_3_ samples at different concentrations.


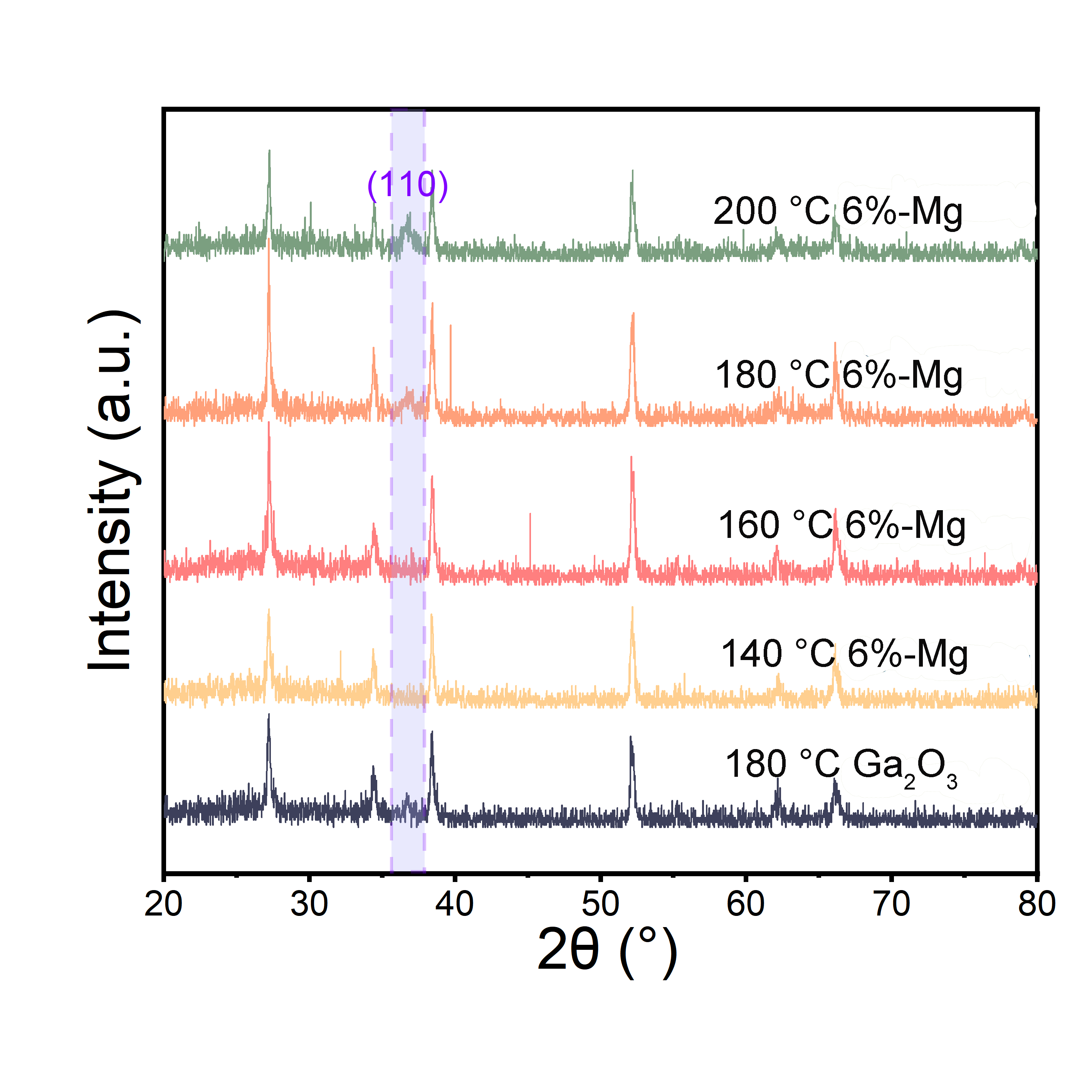


**Figure S9** XRD patterns of Mg-doped α-Ga_2_O_3_ samples under different growth temperatures.


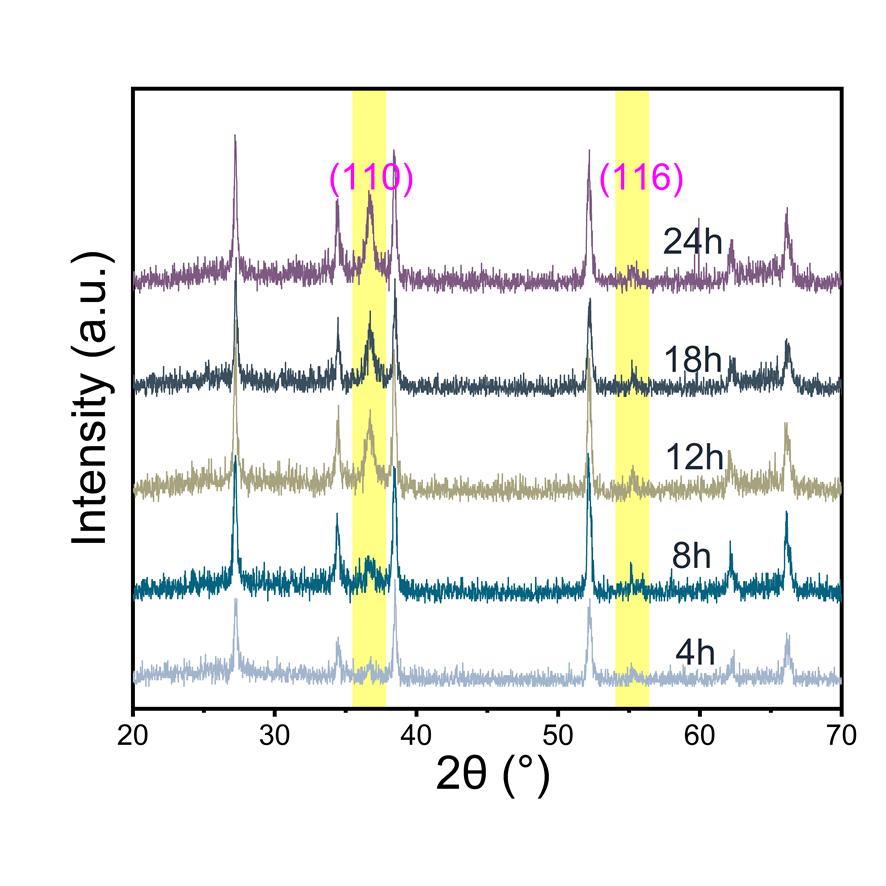


**Figure S10** XRD patterns of Mg-doped α-Ga_2_O_3_ samples under different reaction times.


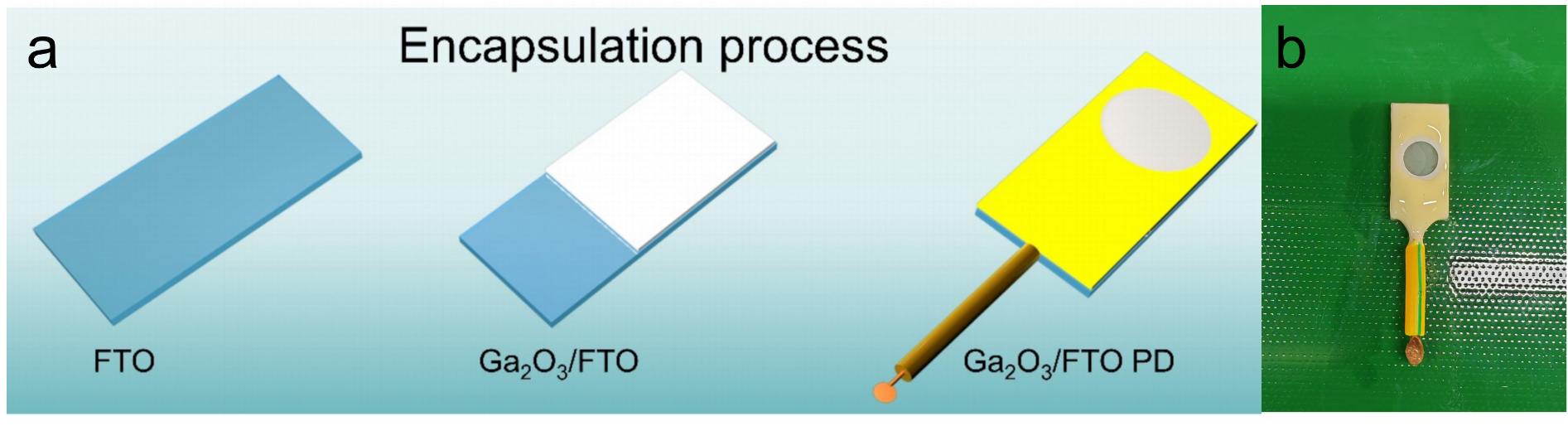
**Figure S11** Schematic diagram and photographs of the light anode encapsulation process for PEC.


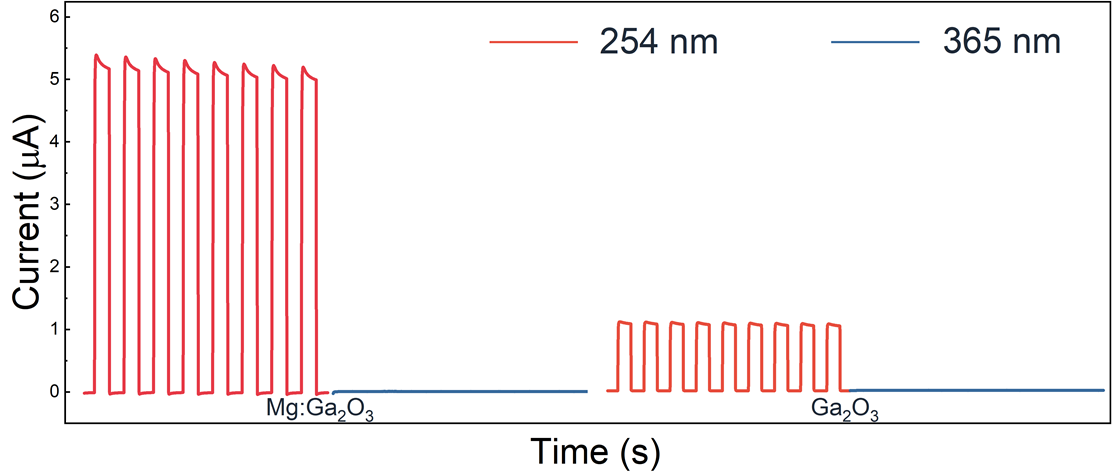


**Figure S12** A comparison of the photocurrents of α-Ga_2_O_3_ and Mg-doped α-Ga_2_O_3_ PEC-PDs under 254/365 nm light.


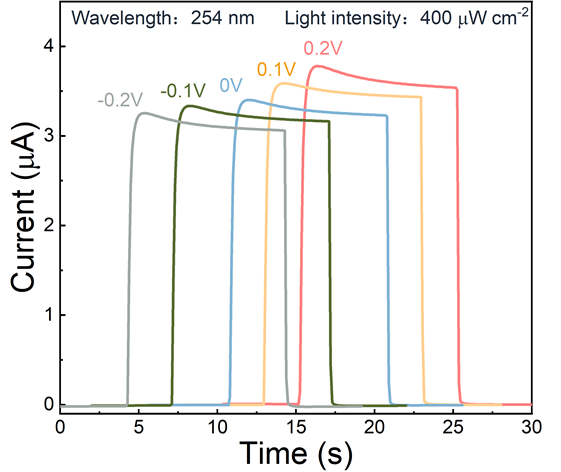


**Figure S13** I-T curves of Mg-doped α-Ga_2_O_3_ PEC-PDs under various bias voltages.


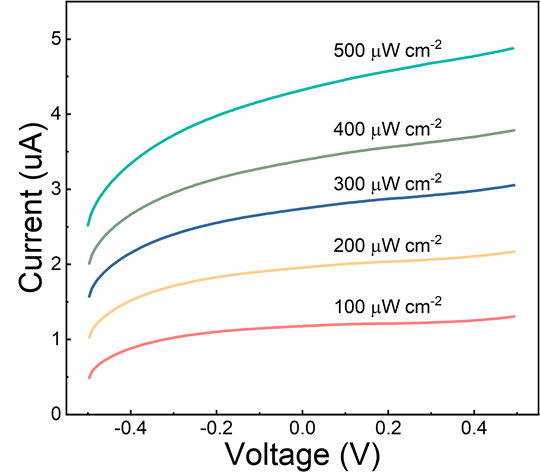


**Figure S14** Comparison of I-V curves of Mg-doped α-Ga_2_O_3_ PEC-PDs under different bias conditions.


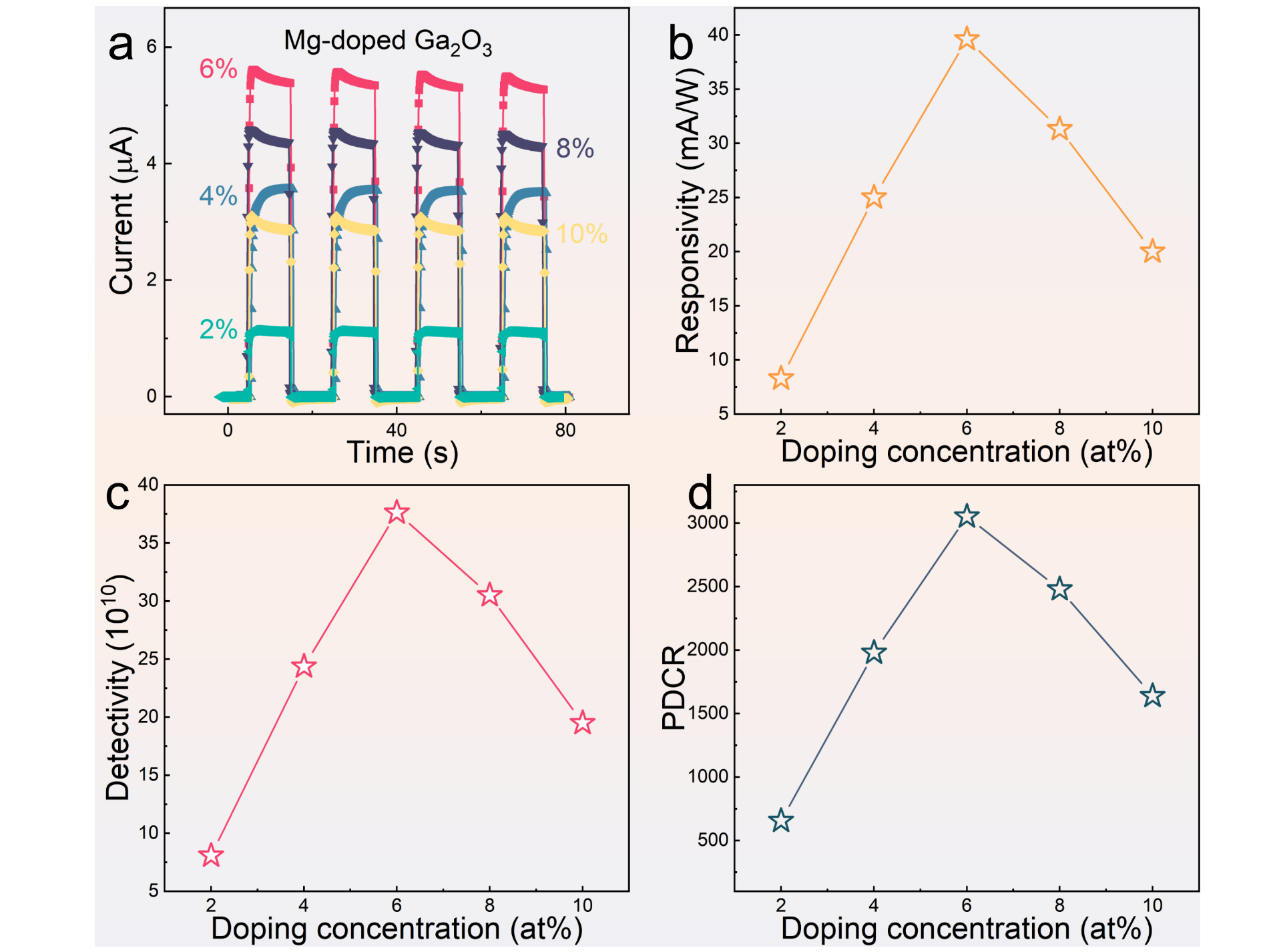


**Figure S15** PEC properties of Mg-doped α-Ga_2_O_3_ samples at different doping concentrations.


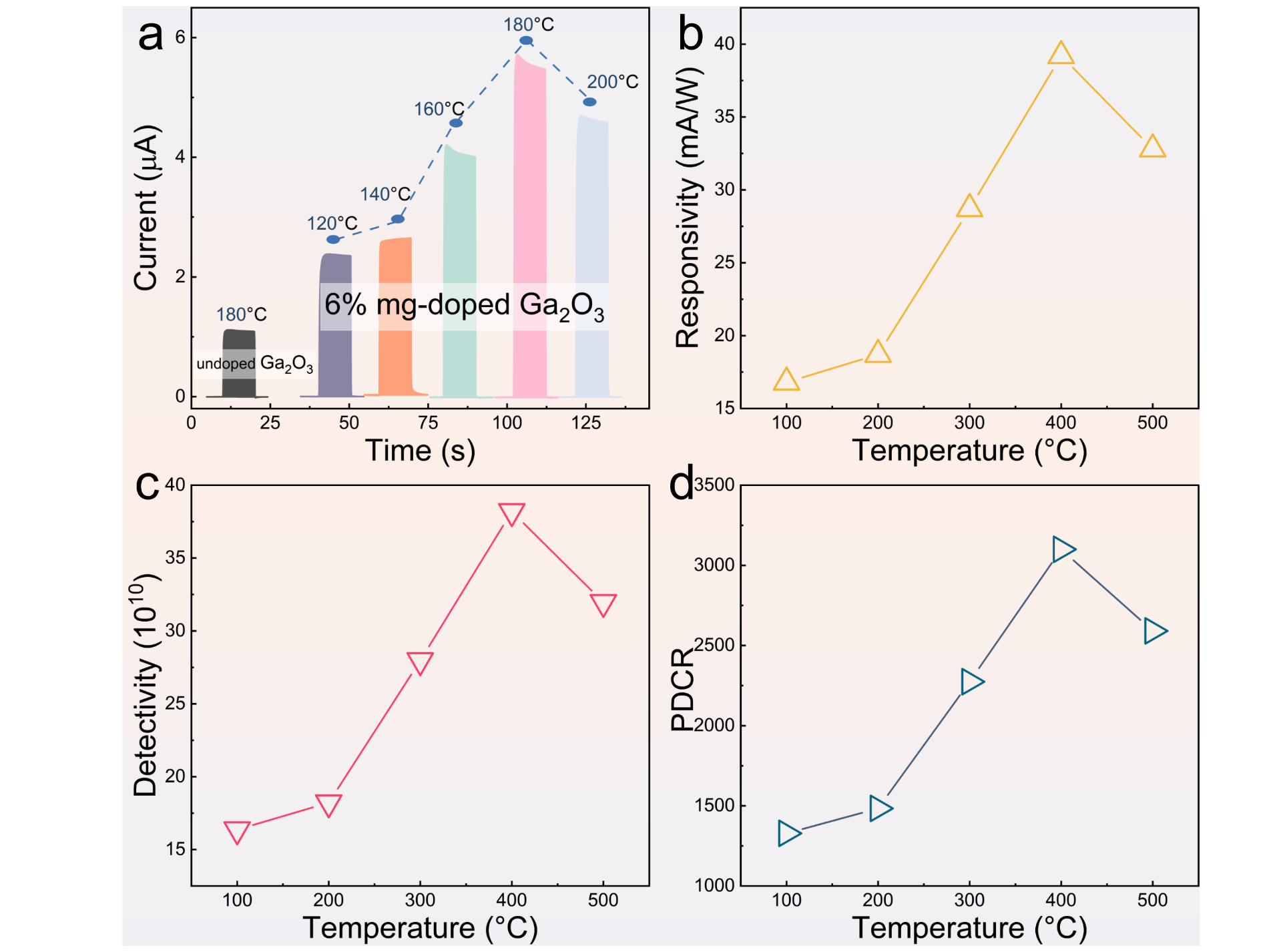


**Figure S16** PEC properties of Mg-doped α-Ga_2_O_3_ samples plotted under different growth temperatures.


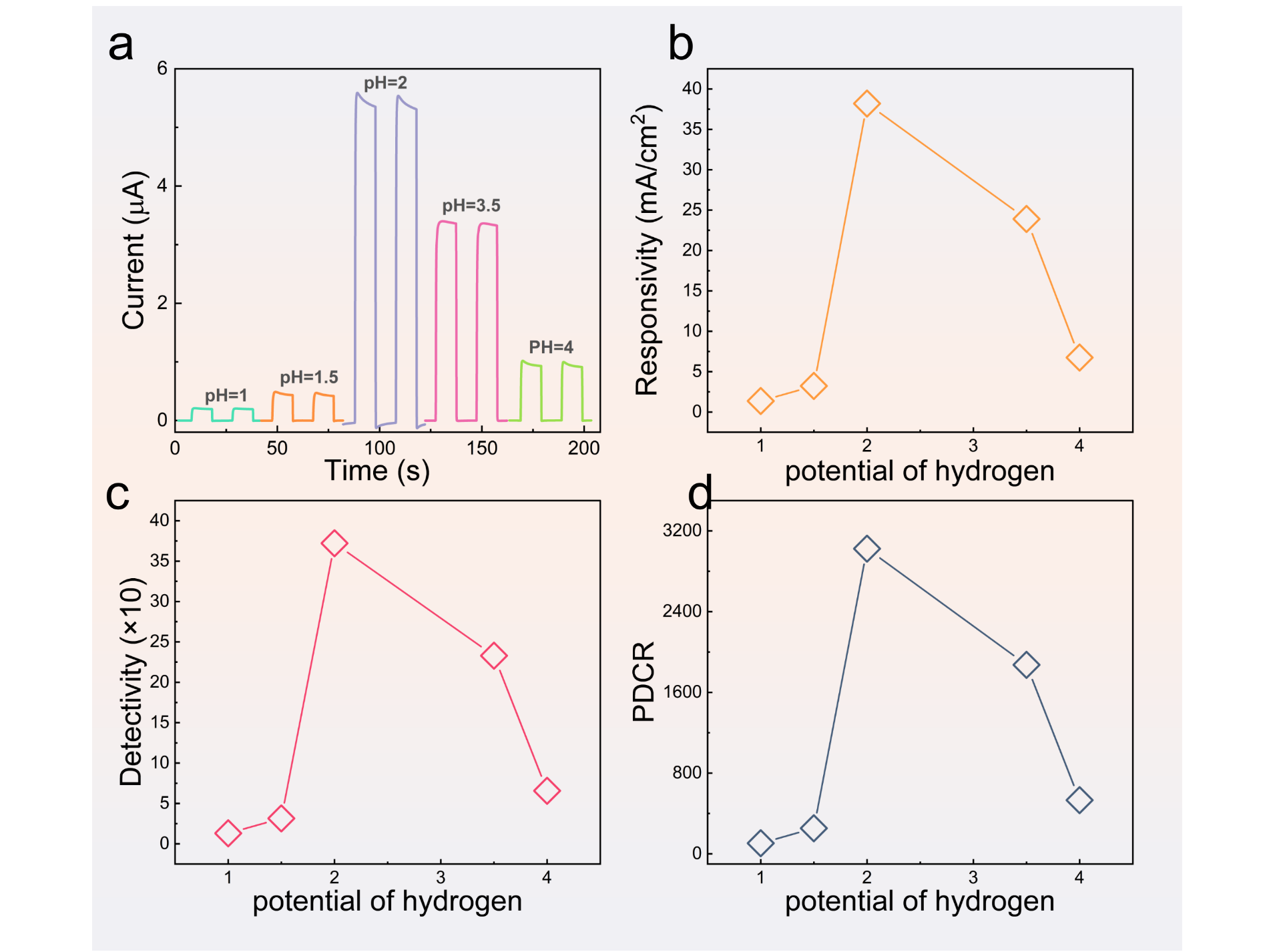


**Figure S17** PEC properties of Mg-doped α-Ga_2_O_3_ samples at different pH.


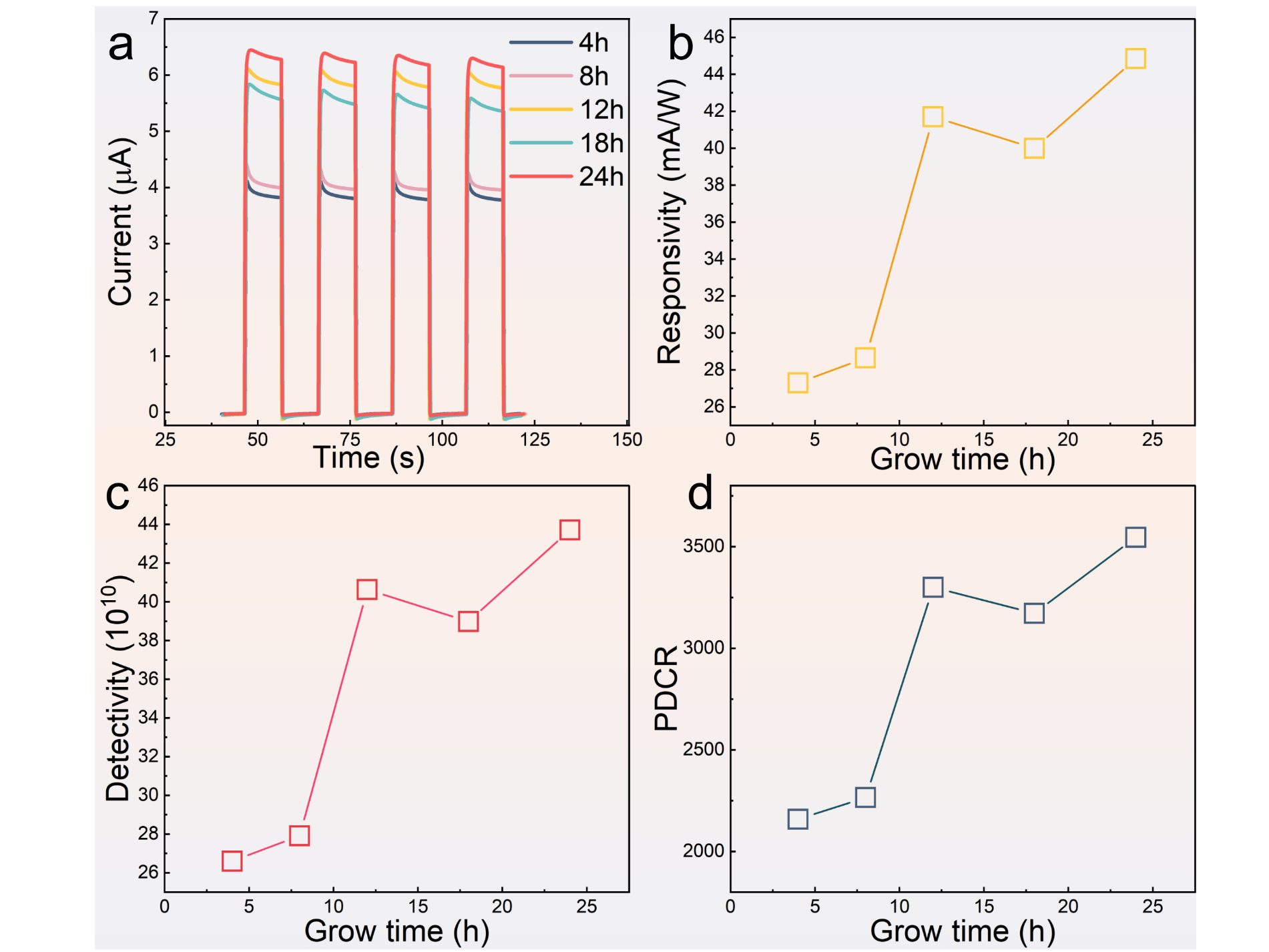


**Figure S18** PEC performance of Mg-doped α-Ga_2_O_3_ samples at different growth times.


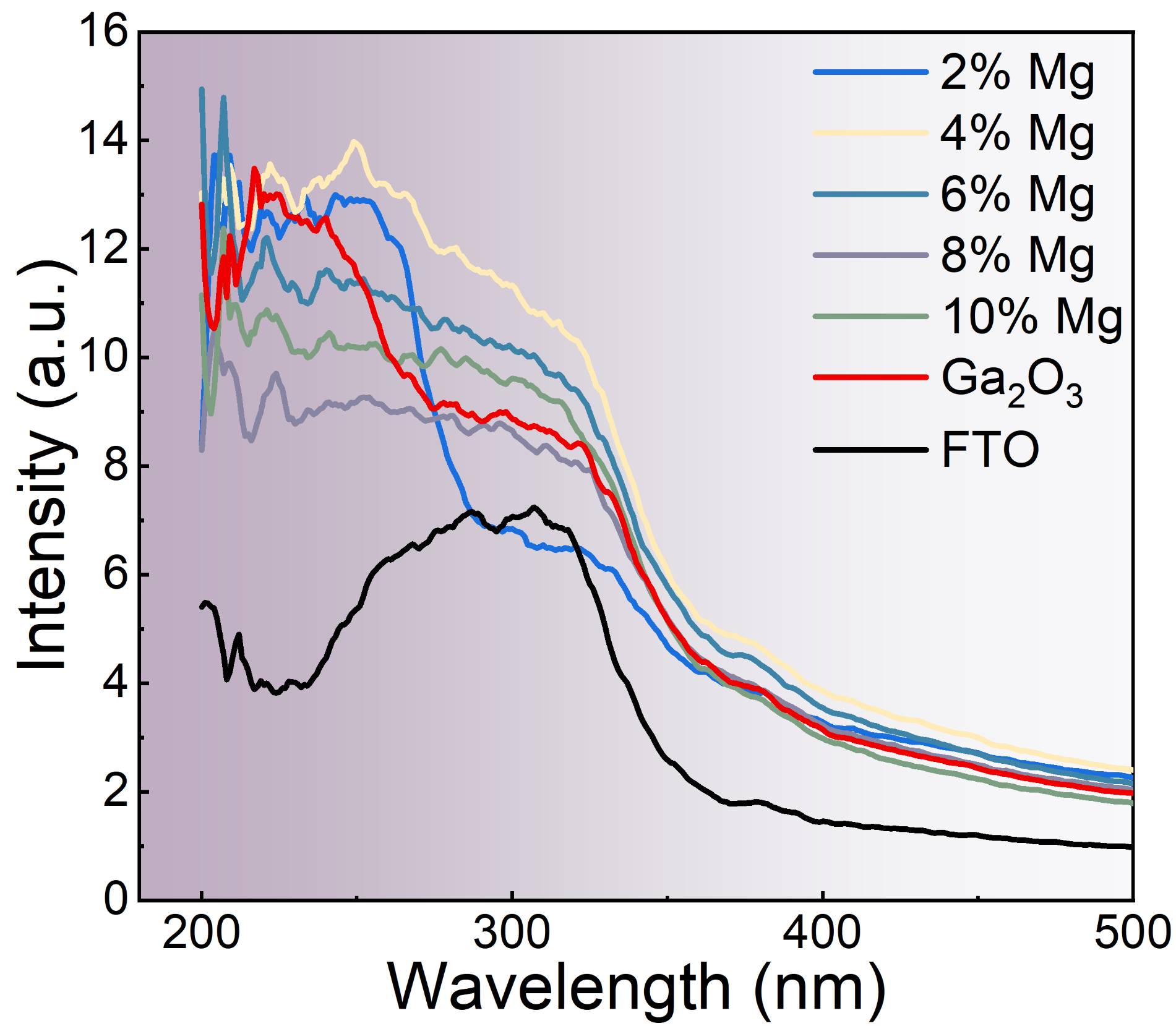


**Figure S19** Absorption of Mg-doped α-Ga_2_O_3_ samples at various doping concentrations.


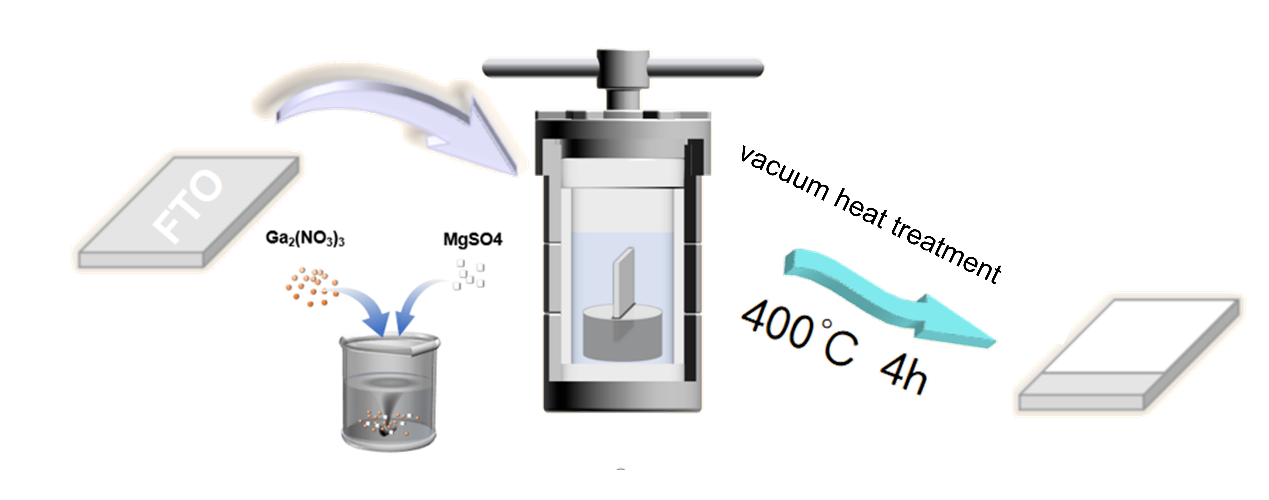


**Figure S20** Schematic illustration of the hydrothermal synthesis process for nanorods preparation.

**Table S1** The synthesis parameters of Mg-doped α-Ga_2_O_3_ nanorods.

| **Temperature** | **Concentration of Mg** | **pH** | **Reaction time (h)** | |
| --- | --- | --- | --- | --- |
| 120℃ | 2% | 1 | 4 |  |
| 140℃ | 4% | 1.5 | 8 |  |
| 160℃ | 6% | 2 | 12 |  |
| 180℃ | 8% | 3.5 | 18 |  |
| 200℃ | 10% | 4 | 24 |  |

**Table S2**. Comparison of different detectors.

| **Material** | **Device type** | **Condition** | **R**  **(mA/W)** | **Detectivity**  **(jones)** | **PDCR** | **Time(s)** | **Ref.** |
| --- | --- | --- | --- | --- | --- | --- | --- |
| **Mg-doped α-Ga_2_O_3_** | PEC | 254 nm/0 V  (0.5 mW/cm^2^) | 34 | 6.4×10^11^ | 2.74×10^3^ | 0.29/0.14 | This work |
| **α-Ga_2_O_3_ NRAs** | PEC | 254 nm/0 V  (3.0 mW/cm^2^) | 3.87 | / | / | 0.23/0.15 | [1] |
| **Al-doped α-Ga_2_O_3_** | PEC | 260 nm/0 V  (0.31 mW/cm^2^) | 1.46 | 1×10^10^ | / | 0.421/0.139 | [2] |
| **Al_2_O_3_/α-Ga_2_O_3_** | PEC | 254 nm/0 V  (0.5 mW/cm^2^) | 22.70 | 2.2×10^11^ | 401 | / | [3] |
| **3D V_O_-Ga_2_O_3_/ZnO** | PEC | 266 nm/0 V | 7.975 | / | 6.9×10^4^ | 0.15/1.1 | [4] |
| **α-Ga_2_O_3_/Cu_2_O QDs** | PEC | 254 nm/0 V  (3.0 mW/cm^2^) | 4.57 | 2.11×10^9^ | 93.4 | 0.81/0.97 | [5] |
| **α-Ga_2_O_3_/Cu_2_O p-n** | PEC | 254 nm/0 V  (2.5 mW/cm^2^) | 0.42 | / | / | 10.3/10.1 | [6] |
| **Mg-doped β-Ga_2_O_3_** | MSM | 254 nm/10 V | 23.8 | / | / | 0.33/0.02 | [7] |
| **Mg-doped ε-Ga_2_O_3_** | MSM | 254 nm/5 V | 77.2 | 2.85×10^12^ | 168 | 1.6/5.4 | [8] |
| **α-Ga_2_O_3_ NRAs** | PEC | 245 nm/0 V  (0.24 mW/cm^2^) | 0.83 | / | / | 0.005/0.029 | [9] |
| **α-Ga_2_O_3_ NRAs** | PEC | 254 nm/0 V  (2.8 mW/cm^2^) | 1.44 | / | / | < 0.8/0.2 | [10] |
| **α-Ga_2_O_3_ NRAs** | PEC | 254 nm/0 V  (0.5 mW/cm^2^) | 11.34 | 2.68×10^11^ | 1.01×10^3^ | 1.51/0.18 | [11] |
| **α-Ga_2_O_3_/Ti wire** | PEC | 230 nm/0 V  (0.1 mW/cm^2^) | 11.2 | / | / | 0.1/0.06  (254 nm) | [12] |
| **Ti/α-Ga_2_O_3_** | PEC | 254 nm/0 V | 15.3 | 13.8 × 10^9^ | / | 0.104/0.077 | [13] |
| **α-Ga_2_O_3_-γ-Al_2_O_3_** | PEC | 254 nm/0 V  (2.0 mW/cm^2^) | 0.17 | / | / | <0.10/0.10 | [14] |
| **α-GaOOH/α-Ga_2_O_3_** | PEC | 250 nm/0 V | 0.29 | / | / | 0.24/0.06 | [15] |
| **a-Ga_2_O_3_/CC** | PEC | 254 nm/0 V  (0.5 mW/cm^2^) | 16.98 | / | / | 0.16/0.10 | [16] |
| **a-Ga_2_O_3_/CFP** | PEC | 254 nm/0 V  (0.1 mW/cm^2^) | 12.90 | / | / | 0.15/0.13 | [17] |
| **a-Ga_2_O_3_@Ag NWs** | PEC | 254 nm/0 V  (0.5 mW/cm^2^) | 11.23 | / | / | 0.07/0.09 | [18] |
| **Pt NP@β-Ga_2_O_3_ /NiO** | heterojunction | 254 nm/0 V  (0.27 mW/cm^2^) | 4.27 | 4.2×10^9^ | / | 0.004/0.007 | [19] |
| **Bi_2_Se_3_/a-Ga_2_O_3_/p-Si** | heterojunction | 254 nm/0 V | 1.38 | 3.22×10^10^ | / | 0.34/0.24 | [20] |

**Reference**

1. Zhang, B.; Wu, H.; Feng, C.; Zhang, Z.; Yu, H.; Zhang, C.; Lin, S.; Xu, C.; Bai, H.; Guo, F., Self-powered solar-blind photodetectors based on α-Ga_2_O_3_ nanorod arrays. *ACS Applied Nano Materials* **2022,** *5* (8), 11956-11963.
2. Guo, J.-C.; Sun, G.-W.; Fan, M.-M.; Fu, X.-C.; Yao, J.-J.; Wang, Y.-D., Hydrothermal Growth of an Al-Doped α-Ga_2_O_3_ Nanorod Array and Its Application in Self-Powered Solar-Blind UV Photodetection Based on a Photoelectrochemical Cell. *Micromachines* **2023,** *14* (7), 1336.
3. Wang, X.; Ding, K.; Huang, L.; Li, X.; Ye, L.; Luo, J.; Jiang, J.; Li, H.; Xiong, Y.; Ye, L., Enhancing the performance of Self-Powered Deep-Ultraviolet photoelectrochemical photodetectors by constructing α-Ga_2_O_3_@a-Al_2_O_3_ Core-Shell nanorod arrays for Solar-Blind imaging. *Applied Surface Science* **2024,** *648*, 159022.
4. Han, P.; Kang, T.; Chen, W.; Gao, M.; Teng, F.; Hu, P.; Fan, H., Cu_2_O quantum dots modified α-Ga_2_O_3_ nanorod arrays as a heterojunction for improved sensitivity of self-powered photoelectrochemical detectors. *Journal of Alloys and Compounds* **2023,** *952*, 170063.
5. Ni, D.; Wang, Y.; Li, A.; Huang, L.; Tang, H.; Liu, B.; Cheng, C., ALD oxygen vacancy-rich amorphous Ga_2_O_3_ on three-dimensional urchin-like ZnO arrays for high-performance self-powered solar-blind photodetectors. *Nanoscale* **2022,** *14* (8), 3159-3165.
6. He, C.; Guo, D.; Chen, K.; Wang, S.; Shen, J.; Zhao, N.; Liu, A.; Zheng, Y.; Li, P.; Wu, Z., α-Ga_2_O_3_ nanorod array–Cu_2_O microsphere p–n junctions for self-powered spectrum-distinguishable photodetectors. *ACS Applied Nano Materials* **2019,** *2* (7), 4095-4103.
7. Qian, Y. P.; Guo, D. Y.; Chu, X. L.; Shi, H. Z.; Zhu, W. K.; Wang, K.; Huang, X. K.; Wang, H.; Wang, S. L.; Li, P. G., Mg-doped p-type β-Ga_2_O_3_ thin film for solar-blind ultraviolet photodetector. *Materials Letters* **2017,** *209*, 558-561.
8. Liu, Z.; Huang, Y.; Li, H.; Zhang, C.; Jiang, W.; Guo, D.; Wu, Z.; Li, P.; Tang, W., Fabrication and characterization of Mg-doped ε-Ga_2_O_3_ solar-blind photodetector. *Vacuum* **2020,** *177*, 109425.
9. Chen, K.; Wang, S.; He, C.; Zhu, H.; Zhao, H.; Guo, D.; Chen, Z.; Shen, J.; Li, P.; Liu, A., Photoelectrochemical self-powered solar-blind photodetectors based on Ga_2_O_3_ nanorod array/electrolyte solid/liquid heterojunctions with a large separation interface of photogenerated carriers. *ACS Applied Nano Materials* **2019,** *2* (10), 6169-6177.
10. Zheng, Z.-Y.; Fan, M.-M., Photoelectrochemical properties of self-powered corundum-structured Ga_2_O_3_ nanorod array/fluorine-doped SnO_2_ photodetectors modulated by precursor concentrations. *Nanotechnology* **2024,** *35* (32), 325702.
11. Huang, L.; Hu, Z.; He, X.; Ma, T.; Li, M.; Zhang, H.; Xiong, Y.; Kong, C.; Ye, L.; Li, H., Self-powered solar-blind ultraviolet photodetector based on α-Ga_2_O_3_ nanorod arrays fabricated by the water bath method. *Optical Materials Express* **2021,** *11* (7), 2089-2098.
12. Chen, Z.; Han, P.; Chen, W.; Wan, Z.; Yang, J.; Liu, Z.; Hu, P.; Teng, F.; Fan, H., Flexible and Self-Powered Solar-Blind UV Photodetector Based on the Ti/α-Ga_2_O_3_/Electrolyte Heterojunction with High Stability. *ACS Applied Electronic Materials* **2023,** *6* (1), 496-504.
13. Wang, G.; Zhang, X.; Li, J.; Bai, Z.; Wu, H.; Jin, M.; Zhou, J.; Xie, E.; Pan, X., A wire-shaped photoanode of the solar-blind photoelectrochemical-type photodetector based on an α-Ga_2_O_3_ nanorods/electrolyte solid/liquid heterojunction. *Applied Physics Letters* **2023,** *122* (7).
14. Zhang, J.; Jiao, S.; Wang, D.; Gao, S.; Wang, J.; Zhao, L., Nano tree-like branched structure with α-Ga_2_O_3_ covered by γ-Al_2_O_3_ for highly efficient detection of solar-blind ultraviolet light using self-powered photoelectrochemical method. *Applied Surface Science* **2021,** *541*, 148380.
15. Zhang, D.; Zhou, X.; Xiong, Y.; Zhang, H.; Ye, L.; Pang, D.; Tang, Y.; Li, H.; Li, W., Flexible self-powered solar-blind UV photodetectors based on amorphous Ga_2_O_3_ modified carbon fiber cloth. *Journal of Alloys and Compounds* **2023,** *969*, 172483.
16. Zhang, Y.; Jiao, S.; Zhang, J.; Liu, S.; Wang, D.; Gao, S.; Wang, J., Study on the evolution from α-GaOOH to α-Ga_2_O_3_ and solar-blind detection behavior of an α-GaOOH/α-Ga_2_O_3_ heterojunction. *CrystEngComm* **2022,** *24* (9), 1789-1794.
17. Huang, L.; Hu, Z.; Zhang, H.; Xiong, Y.; Fan, S.; Kong, C.; Li, W.; Ye, L.; Li, H., A simple, repeatable and highly stable self-powered solar-blind photoelectrochemical-type photodetector using amorphous Ga_2_O_3_ films grown on 3D carbon fiber paper. *Journal of Materials Chemistry C* **2021,** *9* (32), 10354-10360.
18. Yu, C.; Li, H.; Ding, K.; Huang, L.; Zhang, H.; Pang, D.; Xiong, Y.; Yang, P. A.; Fang, L.; Li, W., Flexible and Self‐Powered Photoelectrochemical‐Type Solar‐Blind Photodetectors Based on Ag Nanowires‐Embedded Amorphous Ga_2_O_3_ Films. *Advanced Optical Materials* **2024**, 2400116.
19. Han, Y.; Jiao, S.; Jing, J.; Chen, L.; Rong, P.; Ren, S.; Wang, D.; Gao, S.; Wang, J., A self-powered solar-blind UV-enhanced Bi_2_Se_3_/a-Ga_2_O_3_/p-Si heterojunction photodetector for full spectral photoresponse and imaging. *Nano Research* **2024,** *17* (4), 2960-2970.
20. Yu, J.; Yu, M.; Wang, Z.; Yuan, L.; Huang, Y.; Zhang, L.; Zhang, Y.; Jia, R., Improved photoresponse performance of self-powered β-Ga₂O₃/NiO heterojunction UV photodetector by surface plasmonic effect of Pt nanoparticles. *IEEE Transactions on Electron Devices* **2020,** *67* (8), 3199-3204.
